# Syntheses and crystal structures of the cerium-based coordination polymers poly[(acetic acid)bis­(μ-5-carb­oxy­thio­phene-2-carboxyl­ato)bis­(μ-thio­phene-2,5-di­carboxyl­ato)dicerium(III)] and poly[(μ-acetato)­aqua­(μ_4_-thio­phene-2,5-di­carboxyl­ato)cerium(III)]

**DOI:** 10.1107/S2056989025000210

**Published:** 2025-01-17

**Authors:** Niklas Ruser, Christian Näther, Norbert Stock

**Affiliations:** a Institute of Inorganic Chemistry, Kiel University, Max-Eyth-Str. 2, 24118 Kiel, Germany; Institute of Chemistry, Chinese Academy of Sciences

**Keywords:** synthesis, crystal structure, solvothermal reaction, coordination polymer

## Abstract

The title compounds, [Ce_2_(C_6_H_3_SO_4_)_2_(C_6_H_2_SO_4_)_2_(CH_3_COOH)]_*n*_ (**1**) and [Ce(CH_3_COO)(C_6_H_2_SO_4_)(H_2_O)]_*n*_ (**2**) were synthesized by solvothermal reaction of Ce(NO_3_)_3_·6H_2_O with 2,5-thio­phenedi­carb­oxy­lic acid (H_2_TDC, C_6_H_4_SO_4_) and acetic acid in aceto­nitrile or ethanol/water, respectively. These compounds were obtained as single crystals among other crystalline phases. Both compounds belong to the family of three-dimensional coordination polymers. The asymmetric unit of **1** consists of two Ce^III^ cations, two TDC^2−^ dianions, two HTDC^−^ anions and one acetic acid ligand, all of them located in general positions. The two Ce^III^ cations are each coordinated by eight oxygen atoms in the form of distorted square anti­prisms. These are bridged by carboxyl­ate and carb­oxy­lic acid groups, forming mono-periodic hybrid inorganic building units (IBUs), which are connected into a three-dimensional network by the TDC^2−^ and HTDC^−^ ligands. Compound **2** has an asymmetric unit composed of one Ce^III^ cation, one TDC^2−^ dianion, one acetate anion and one water mol­ecule in general positions. The cerium atom is eightfold coordinated by oxygen atoms. Edge-sharing CeO_8_ polyhedra form a dinuclear IBU (Ce_2_O_14_). This IBU is bridged by six TDC^2−^ linkers to nine other IBUs, forming a three-dimensional framework. Both title compounds exhibit additional O—H⋯O hydrogen bonds. Powder X-ray diffraction shows that both compounds were not obtained as pure phases.

## Abstract 2

In the crystal structures of the title compounds, the Ce^III^ cations are eightfold coordinated and linked into three-dimensional frameworks by ligands based on different deprotonated derivates of 2,5-thio­phenedi­carb­oxy­lic acid. Both compounds exhibit O—H⋯O hydrogen bonds.

## 1. Chemical context

The search for new coordination polymers (CPs) (Batten *et al.*, 2009 ▸), especially metal–organic frameworks (MOFs) (Rowsell & Yaghi, 2004 ▸; Long & Yaghi, 2009 ▸) is still a very active field of research. As a result of the variety of possible metals, as well as inorganic and organic linker mol­ecules, there are many opportunities to discover new compounds and to modify properties and structural behaviors of existing ones. CPs contain metal atoms/ions linked by ligands that extend in one, two or three dimensions. MOFs are a subclass of CPs that exclusively contain organic ligands, called linkers, that lead to the formation of two- or three-periodic networks. In addition, MOFs have potential pores (Batten *et al.*, 2013 ▸). As a result of their porosity and large specific surface areas, MOFs have potential applications in areas such as catalysis (Hu *et al.*, 2018 ▸; Li, 2018 ▸; Lammert *et al.*, 2015 ▸), gas storage (Li *et al.*, 2019 ▸; Sahayaraj *et al.*, 2023 ▸) and sensing (Shekhah *et al.*, 2011 ▸; Wang *et al.*, 2018 ▸). The properties of MOFs can often be tuned by the selection of metal ions and organic linkers (Sahayaraj *et al.*, 2023 ▸). In the case of cerium MOFs, their redox properties can be exploited in catalytic applications (Lammert *et al.*, 2015 ▸; Smolders *et al.*, 2018 ▸, 2020 ▸).
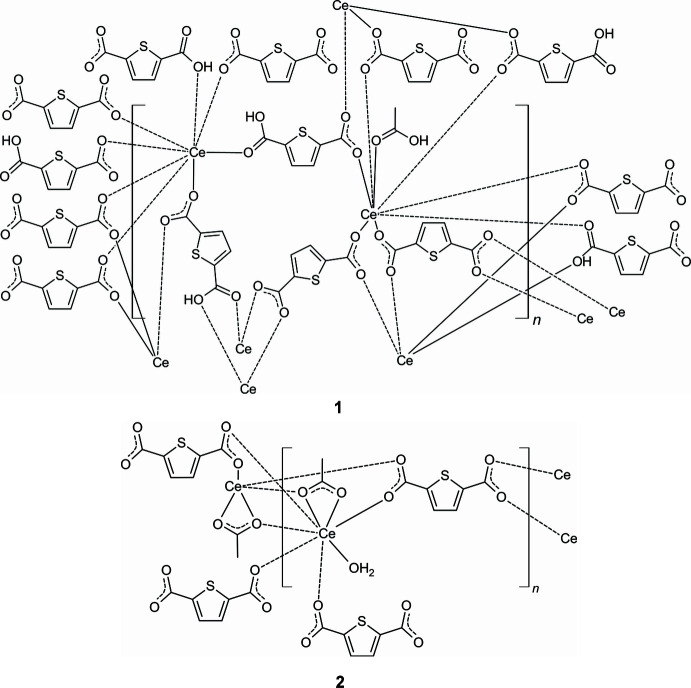


The variety of possible reactants is matched by the variety of synthesis conditions under which these compounds can be obtained. Therefore, there are multidimensional parameter spaces to explore, which could lead to the discovery of an enormous number of new compounds. A very useful tool for the screening of large parameter spaces is high-throughput methods (Stock, 2010 ▸). High-throughput methods make use of the concepts of automation, parallelization and miniaturization. Many syntheses can be carried out under the same temperature-time program, while the influence of molar ratios of starting materials, solvent mixtures, concentration, *etc*. on the product formation can be studied simultaneously. Phase mixtures are often observed and high-throughput methods can also be used to optimize reaction conditions. In a systematic study of the chemical system Ce^III^/2,5-thio­phenedi­carb­oxy­lic acid (H_2_TDC) in different solvents two new CPs were discovered and structurally characterized by single crystal X-ray diffraction.

## 2. Structural commentary

**Crystal structure of 1**

**Figure 1:**
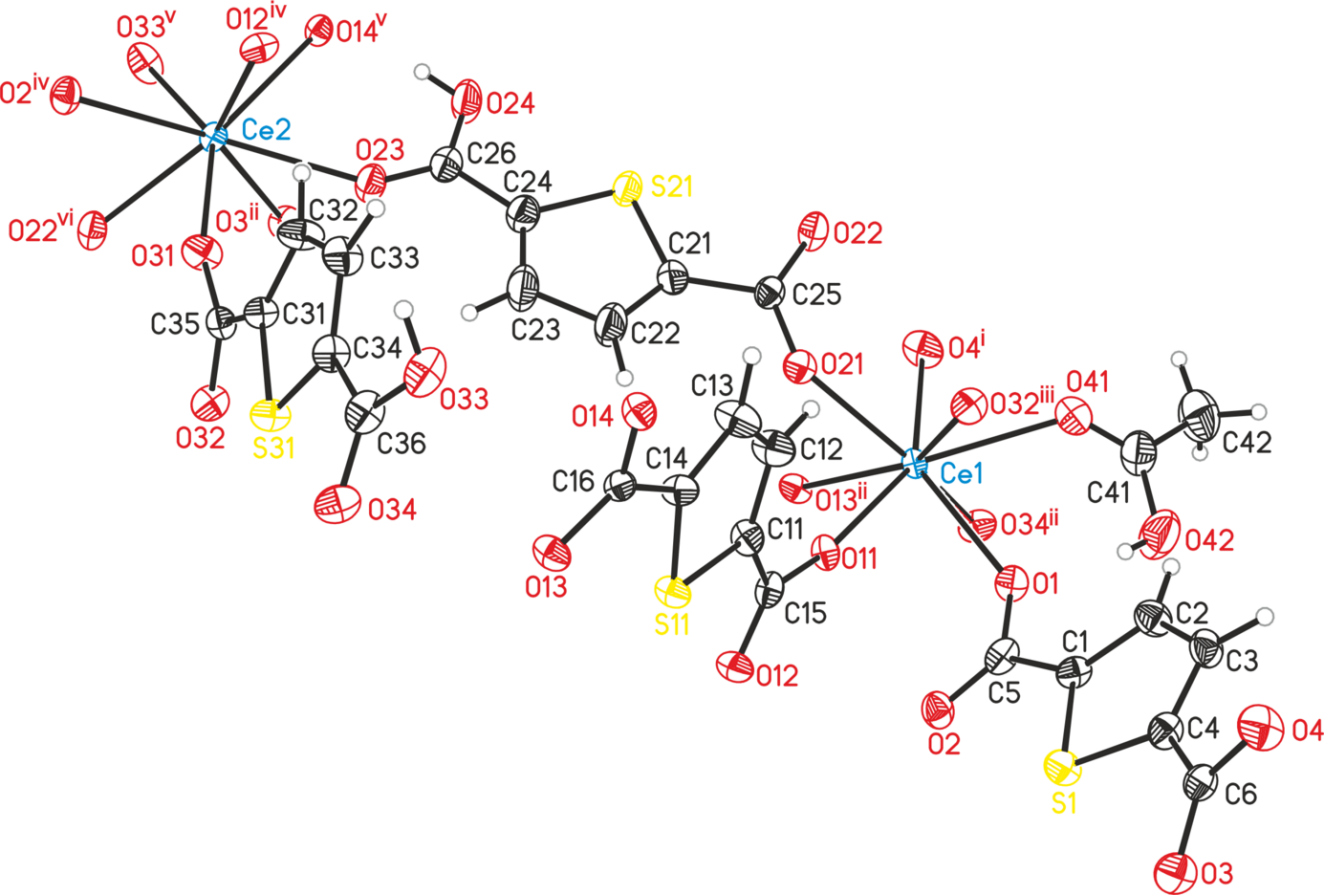
Crystal structure of compound **1** with atom labeling and displacement ellipsoids drawn at the 50% probability level. The HTDC^−^ and TDC^2−^ fragments belonging to the oxygen atoms created by symmetry operations are omitted for clarity. Symmetry codes: (i) −*x* + 1, −*y* + 2, *z* − 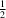; (ii) −*x* + 1, −*y* + 1, *z* − 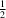; (iii) *x*, *y* + 1, *z*; (iv) *x* − 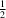, −*y* + 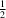, *z*; (v) −*x* + 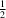, *y* − 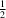, *z* − 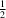; (vi) *x*, *y* − 1, *z*.

**Figure 2:**
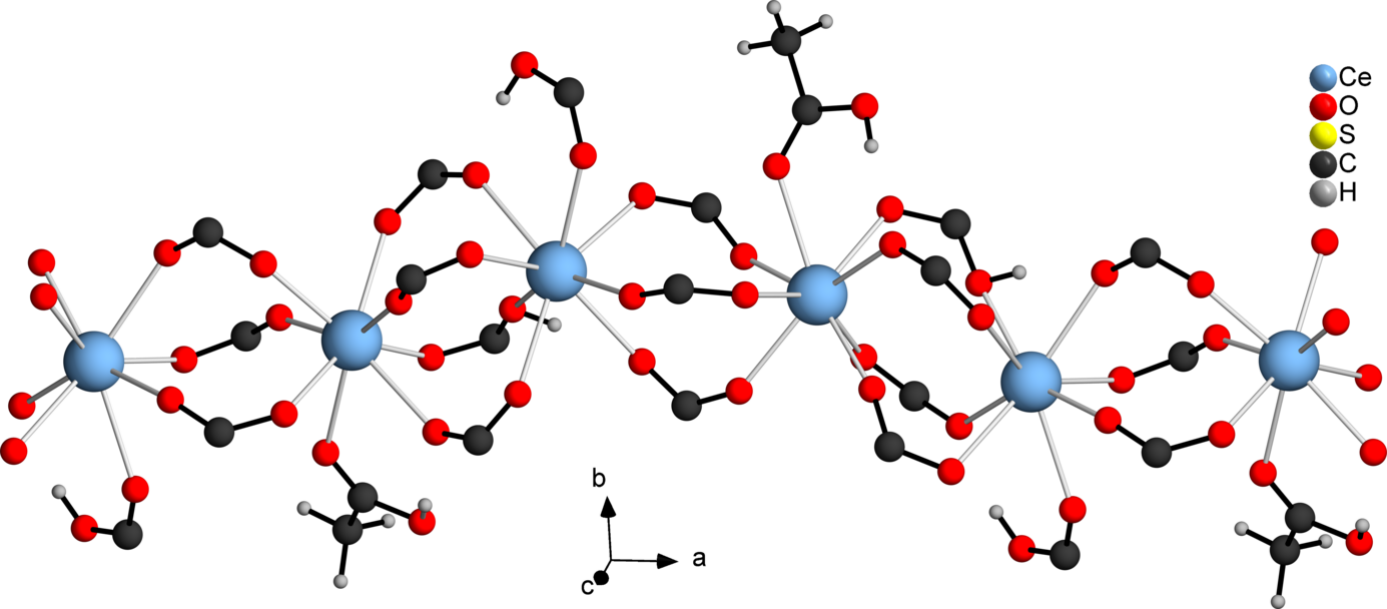
Monoperiodic chain of **1** composed of Ce^III^ cations, carboxyl­ate groups and carb­oxy­lic acid groups.

**Figure 3:**
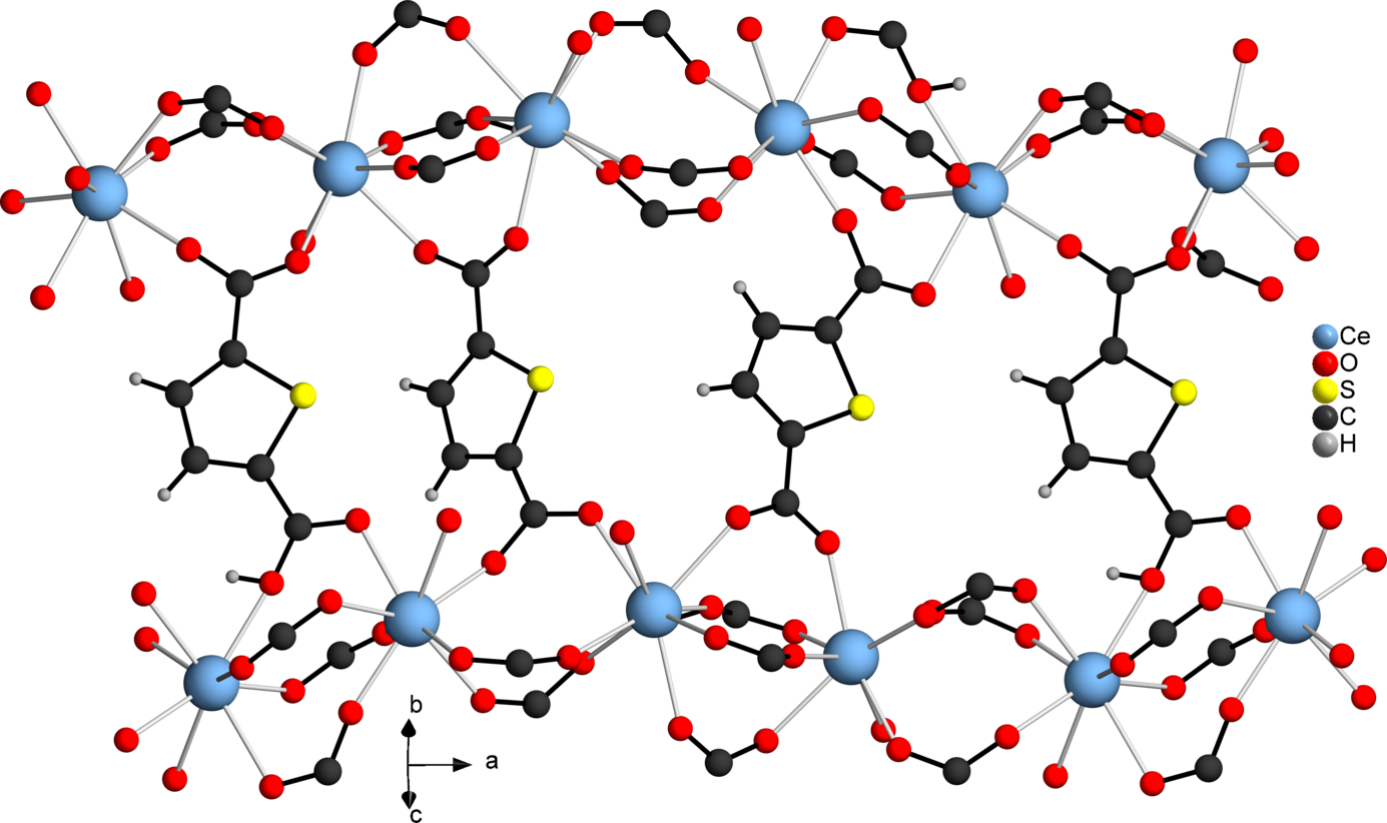
Connection of the monoperiodic chains of **1** by the TDC^2−^ and HTDC^−^ anions.

**Figure 4:**
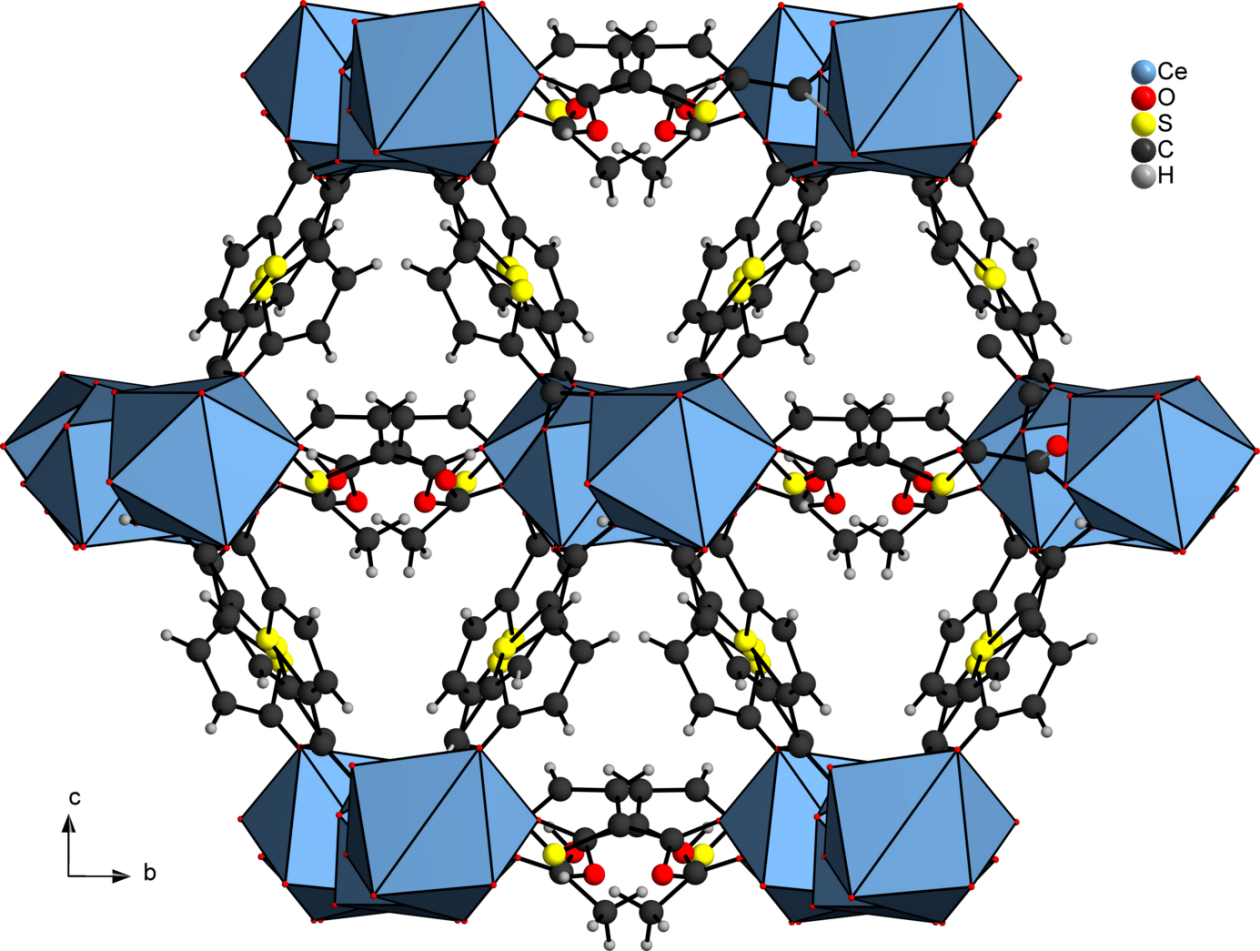
Crystal structure of **1** along the crystallographic *a*-axis. The CeO_8_ units are represented by polyhedra.

The asymmetric unit of [Ce_2_(C_6_H_3_SO_4_)_2_(C_6_H_2_SO_4_)_2_(CH_3_COOH)]_*n*_ (**1**) contains two Ce^III^ cations, two HTDC^−^ anions, two TDC^2−^ dianions and one acetic acid mol­ecule, all located in general positions (Fig. 1 ▸). Both Ce^III^ cations (Ce1 and Ce2) are coordinated by eight oxygen atoms, forming distorted square anti­prisms. These oxygen atoms coordinating Ce1 belong to four TDC^2−^ dianions, three HTDC^−^ anions and one acetic acid mol­ecule. For Ce2, the oxygen atoms originate from four TDC^2−^ dianions and four HTDC^−^ anions. While the binding mode of the carboxyl­ate groups is exclusively bridging μ_2_-(*O*,*O*′), the binding modes of the carb­oxy­lic acid groups of HTDC^−^ are monodentate μ_1_-(*O*) and bridging μ_2_-(*O*,*O*′). The carb­oxy­lic acid group of acetic acid exhibits the monodentate μ_1_-(*O*) binding mode. The Ce—O distances are in the range 2.259 (5)–2.555 (4) Å (Table 1 ▸). The binding modes of the ligands lead to an IBU consisting of CeO_8_ polyhedra, which are bridged by carboxyl­ate and carb­oxy­lic acid groups of TDC^2−^ and HTDC^−^ anions, forming a monoperiodic chain extending along the crystallographic *a*-axis direction (Fig. 2 ▸). Each chain is inter­connected to six other chains by TDC^2−^ and HTDC^−^ anions (Fig. 3 ▸), resulting in a hexa­gonal arrangement of the chains (Fig. 4 ▸). This gives rise to a CP with a three-dimensional network. The acetic acid does not participate in the formation of the network but completes the coordination sphere of Ce1.

**Crystal structure of 2**

**Figure 5:**
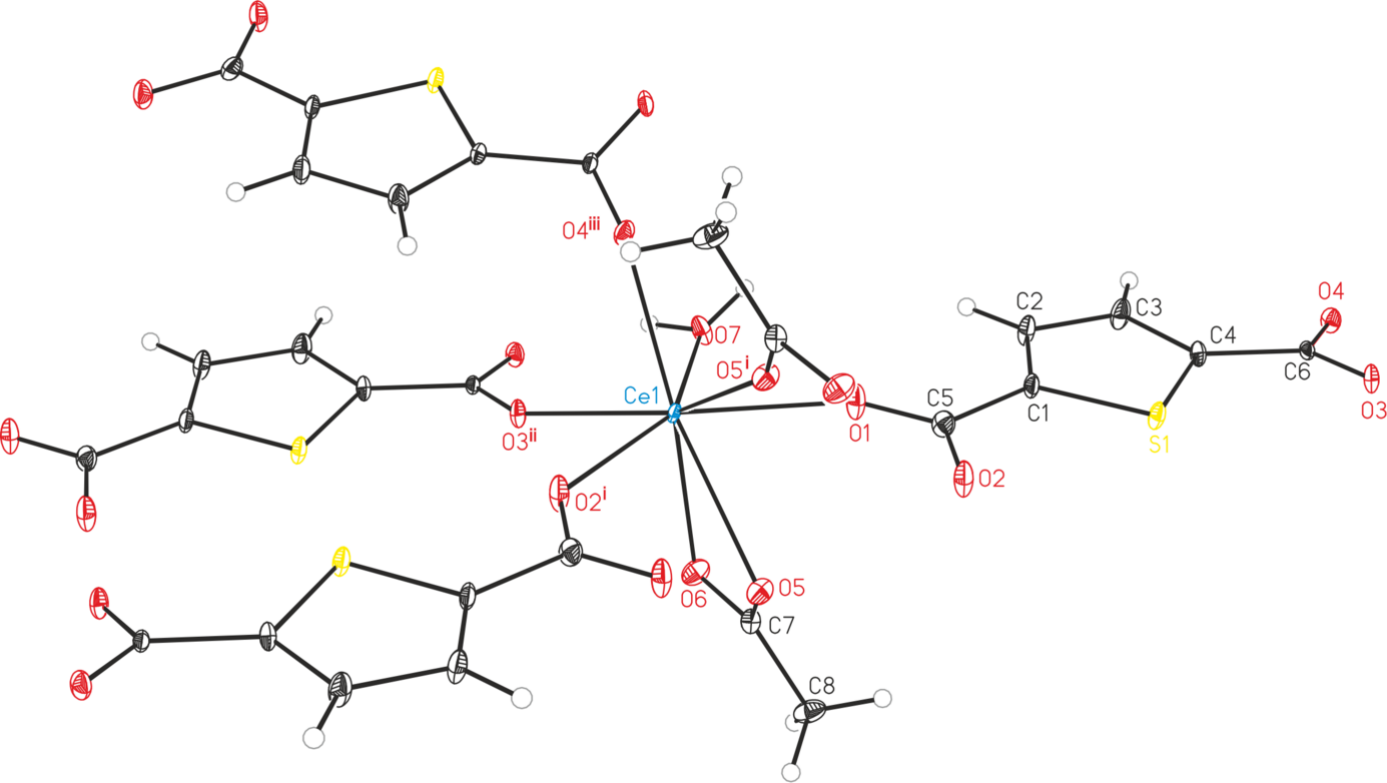
Crystal structure of compound **2** with atom labeling and displacement ellipsoids drawn at the 50% probability level. Symmetry codes: (i) −*x* + 1, −*y* + 1, −*z* + 1; (ii) *x* − 1, *y*, *z* − 1; (iii) *x* − 1, −*y* + 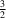, *z* − 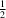.

**Figure 6:**
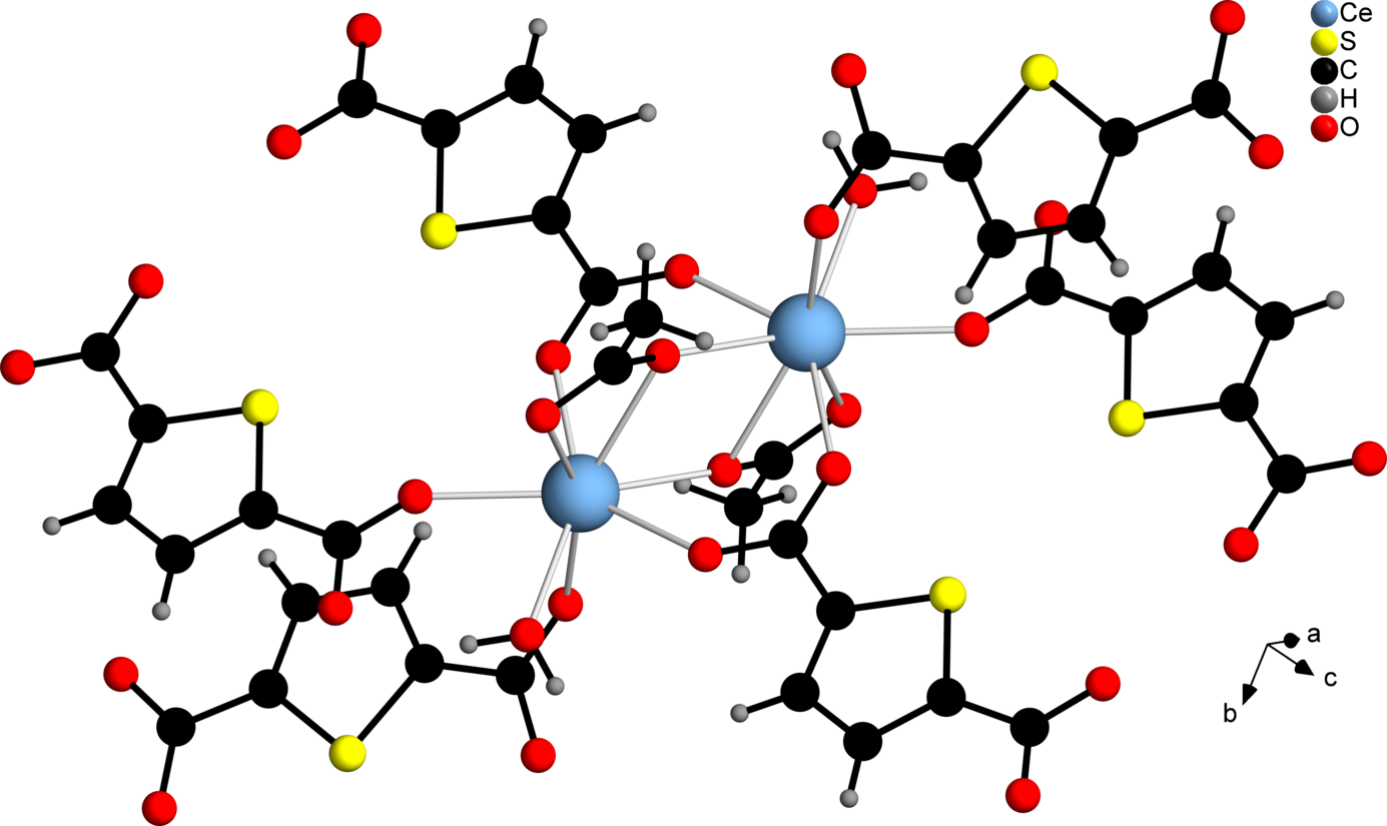
Connection of the CeO_8_ polyhedra and the coordination of the linker mol­ecules to the Ce_2_O_14_ IBU of **2**.

**Figure 7:**
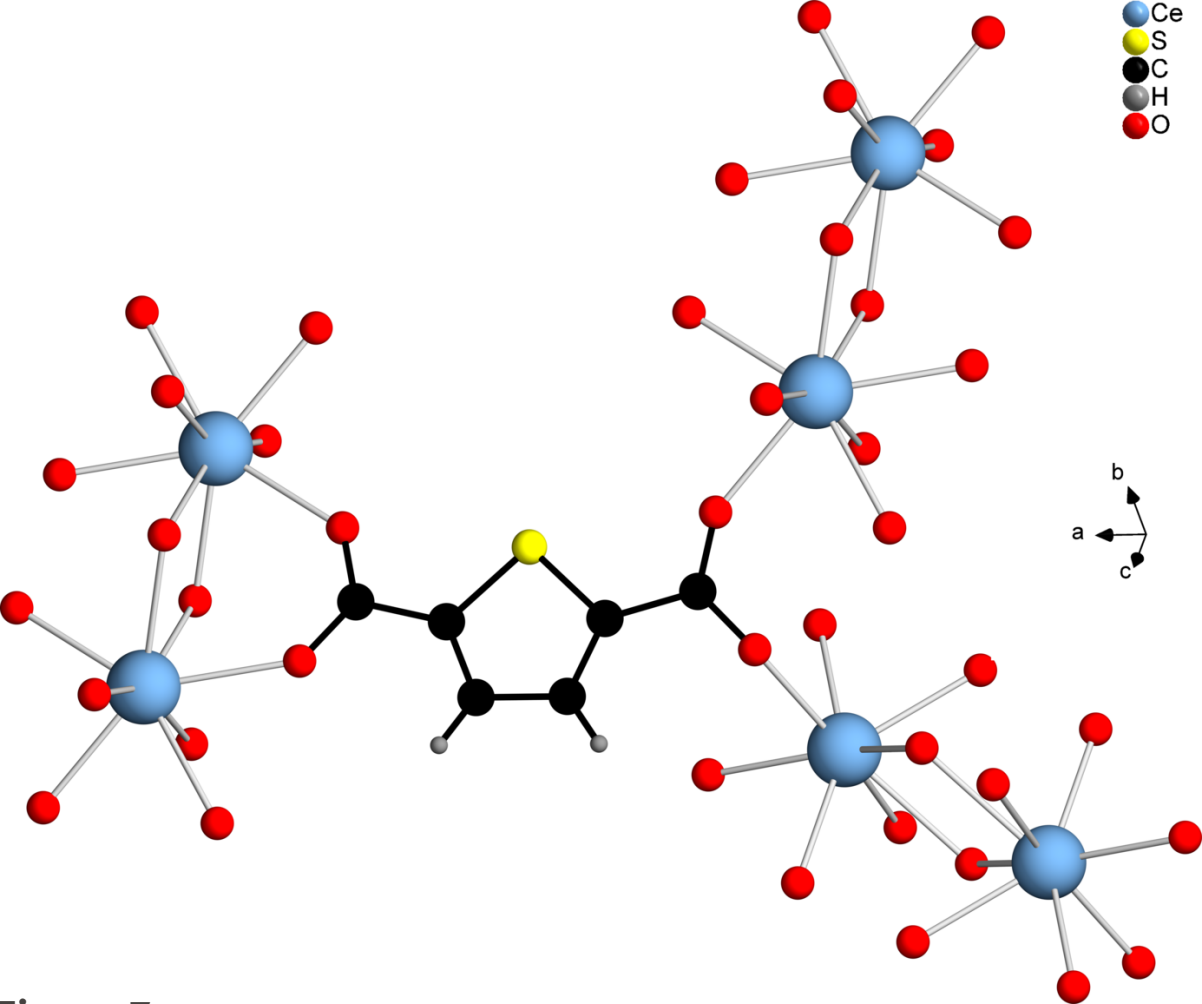
Bridging of three Ce_2_O_14_ IBUs by one TDC^2−^ dianion in **2**.

**Figure 8:**
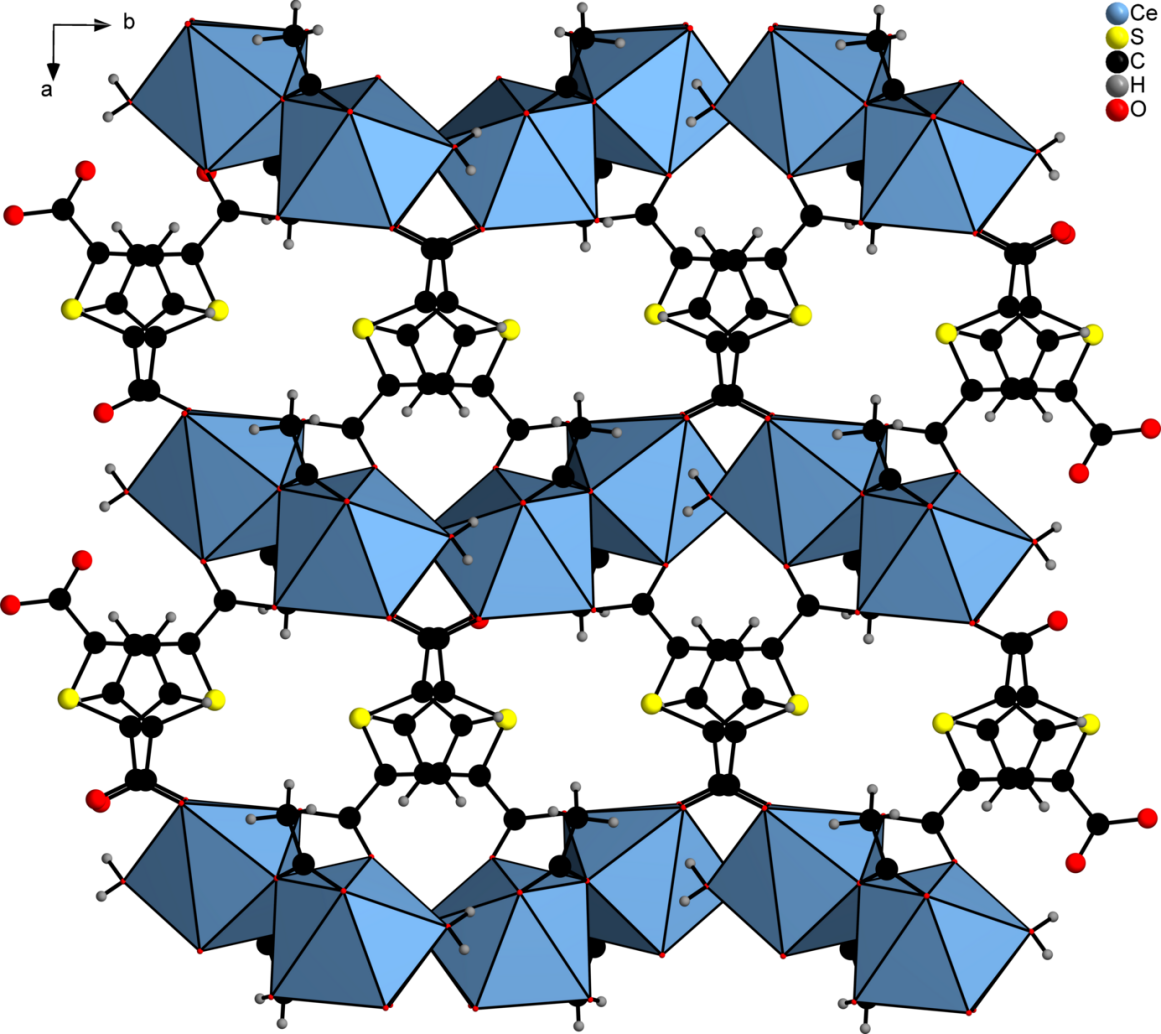
Crystal structure of **2** along the crystallographic *c*-axis. The Ce_2_O_14_ IBUs are represented by polyhedra.

The asymmetric unit of [Ce(C_6_H_2_SO_4_)(CH_3_COO)(H_2_O)]_*n*_ contains one Ce^III^ cation, one CH_3_COO^−^ anion, one TDC^2−^ dianion and one water mol­ecule, all of which are located in general positions (Fig. 5 ▸). The Ce^III^ cation is eightfold coordinated by oxygen atoms that originate from four TDC^2−^ dianions, two acetate ions and one water mol­ecule, forming a distorted square anti­prism. The carboxyl­ate groups of the TDC^2−^ dianions coordinate the Ce^III^ cations exclusively in the bridging μ_2_-(*O*,*O*′) binding mode, while for the acetate ions the bridging chelate μ_2_-(*O*,*O*′,*O*′) binding mode is observed. The Ce—O distances are in the range 2.406 (2)–2.621 (2) Å (Table 2 ▸). Edge-sharing of the CeO_8_ polyhedra leads to a dinuclear Ce_2_O_14_ IBU. Each IBU is surrounded by six TDC^2−^ ligands (Fig. 6 ▸), which connect it to nine other IBUs, while each linker bridges three IBUs (Fig. 7 ▸). This gives rise to a CP with a three-dimensional network (Fig. 8 ▸).

## 3. Supra­molecular features

**Supra­molecular features of 1**

**Figure 9:**
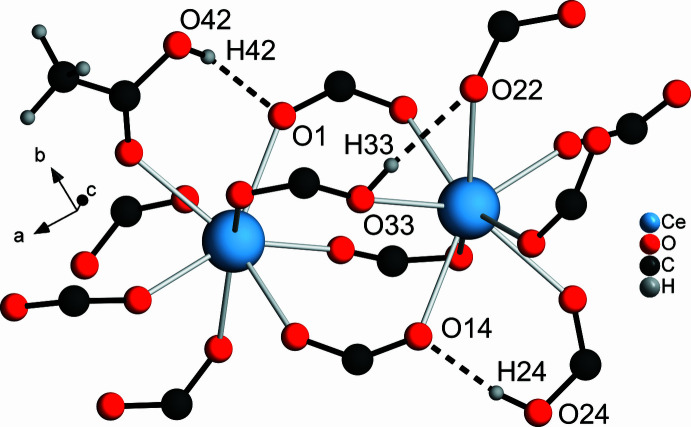
Hydrogen bonds in **1**. The carb­oxy­lic acid groups and carboxyl­ate are related to HTDC^−^ anions and TDC^2−^ dianions, which are not shown completely for the sake of clarity. The hydrogen bonding is shown with dashed lines.

Additionally, **1** exhibits hydrogen bonds (O—H⋯O) between HTDC^−^ and TDC^2−^ anions (O33—H33⋯O22 and O24—H24⋯O14) as well as acetic acid and TDC^2−^ (O42—H42⋯O1, Fig. 9 ▸). The H⋯O distances and O–H⋯O angles (Table 3 ▸) indicate the presence of strong hydrogen bonds (1.5–2.2 Å, 130–180°; Desiraju & Steiner, 1999 ▸). In addition, C—H⋯O hydrogen bonds are found, indicating the presence of weak hydrogen bonds.

**Supra­molecular features of 2**

**Figure 10:**
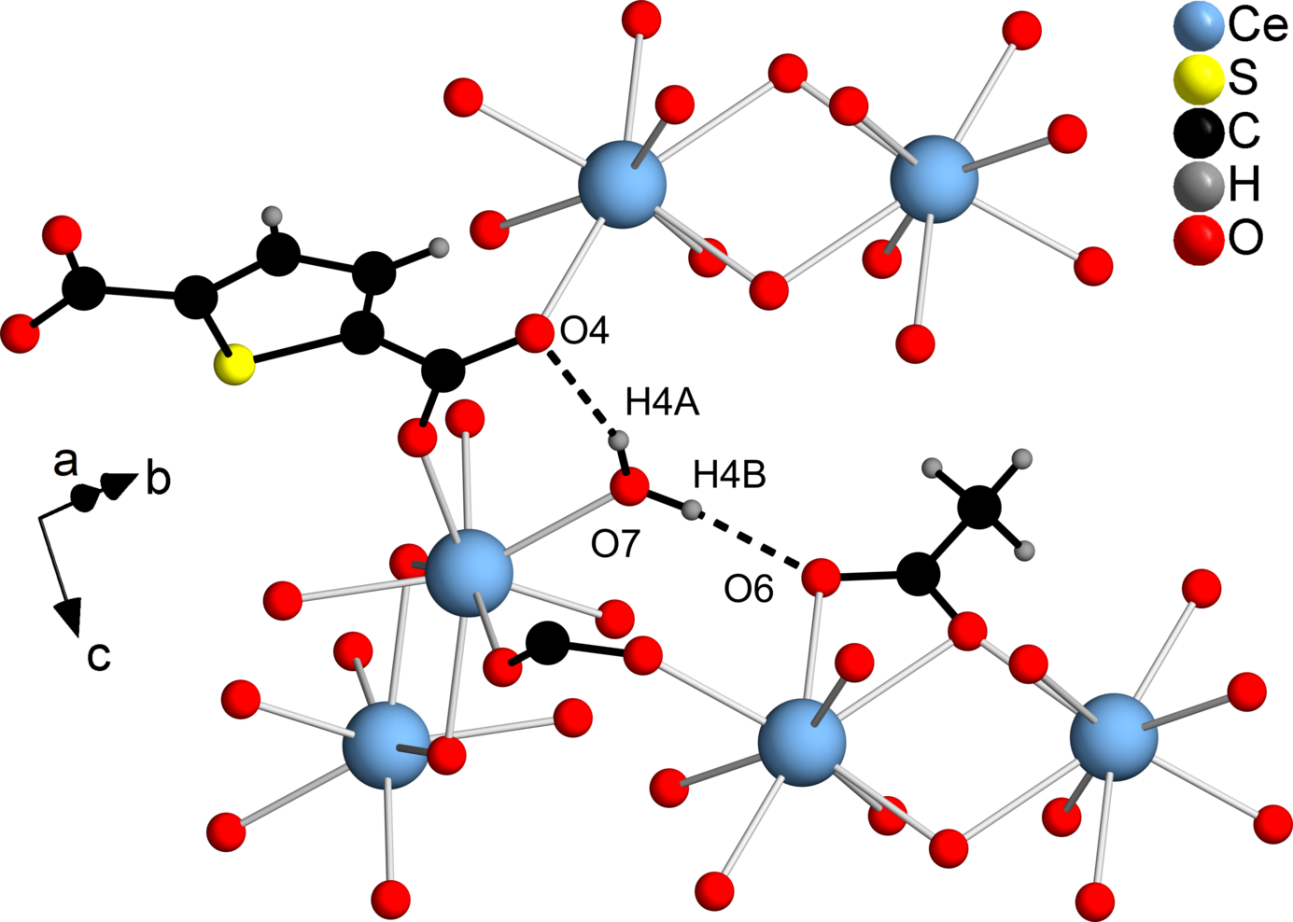
Hydrogen bonds in **2**. The bridging carboxyl­ate group is related to a TDC^2−^ dianion, which was omitted for clarity. The hydrogen bonding is shown with dashed lines.

In **2**, hydrogen bonds (O—H⋯O) are present between the hydrogen atoms of the water mol­ecules and the TDC^2−^ (O7—H4*A*⋯O4) and the acetate anions (O7—H4*B*⋯O6). The presence of these hydrogen bonds leads to the stabilization of the network through the connection with two adjacent IBUs (Fig. 10 ▸). The H⋯O distances and O—H⋯O angles (Table 4 ▸) indicate the presence of strong hydrogen bonds (1.5–2.2 Å, 130–180°; Desiraju & Steiner, 1999 ▸). For C—H⋯O hydrogen bonds, only weak inter­actions can be assumed.

## 4. Database survey

A search for crystal structures containing Ce and TDC^2−^ anions in the Cambridge Structural Database (CSD version 5.45, last update September 2024; Groom *et al.*, 2016 ▸) revealed two structures containing Ce^III^ cations, namely [Ce_6_(TDC)_9_(*N*,*N*-di­ethyl­formamide)_5_(H_2_O)_3_]_*n*_ (refcode UMEKUU; Yawer *et al.*, 2016 ▸) and [Ce_2_(TDC)_3_(*N*,*N*′-di­methyl­acetamide)_2_(H_2_O)]_*n*_ (VUZNEM; Kumar *et al.*, 2020 ▸). Extending the search to any lanthanide cation except Ce results in 150 hits. Restricting the search to the cell parameters of **1** (reduced cell, 5% tolerance) yields nine hits with eight unique compounds, none of which is isostructural to **1**. Restricting the search to the cell parameters of **2** (reduced cell, 5% tolerance) gives 24 hits with 16 unique compounds. Eight of the compounds have the composition [*Ln*(TDC)(CH_3_COO)(H_2_O)]_*n*_ (*Ln* = Pr, Nd, Eu, Gd, Tb, Dy) (refcodes: KILCAL, KILCEP, KILCIT, KILCOZ, KILCUF, KILDAM (Ren *et al.*, 2012 ▸), KILCUF01 (Yuan *et al.*, 2022 ▸) (deposited without coordinates) and KILCUF02 (Han *et al.*, 2024 ▸)], which are isostructural to **2**. Another six results are published as [*Ln*(TDC)(CH_3_COO)(H_2_O)]_*n*_ (*Ln* = Sm, Eu, Gd, Tb, Dy) and are also isostructural to **2** [refcodes: FEFXEF, FEFWIZ, FEFWUL, FEFWOF, FEFXAS (Han *et al.*, 2017 ▸) and FEFWIZ01 (Han *et al.*, 2020 ▸)]. Another ten results are for compounds structurally related to **2** with acetate anions being replaced by nitrate anions [*Ln*(TDC)(NO_3_)(H_2_O)]_*n*_ (*Ln* = Nd, Sm, Eu, Gd, Tb, Dy, Ho, Er, Yb) [refcodes: ICOREZ, ICORID, ICOROJ, ICORUP, ICOSAW, ICOSEA, ICOSIE, ICOSOK, ICOSUQ (Sun *et al.*, 2011) and ICOROJ01 (Adcock *et al.*, 2018 ▸)].

## 5. Synthesis and crystallization

**Synthesis of 1**

**Figure 11:**
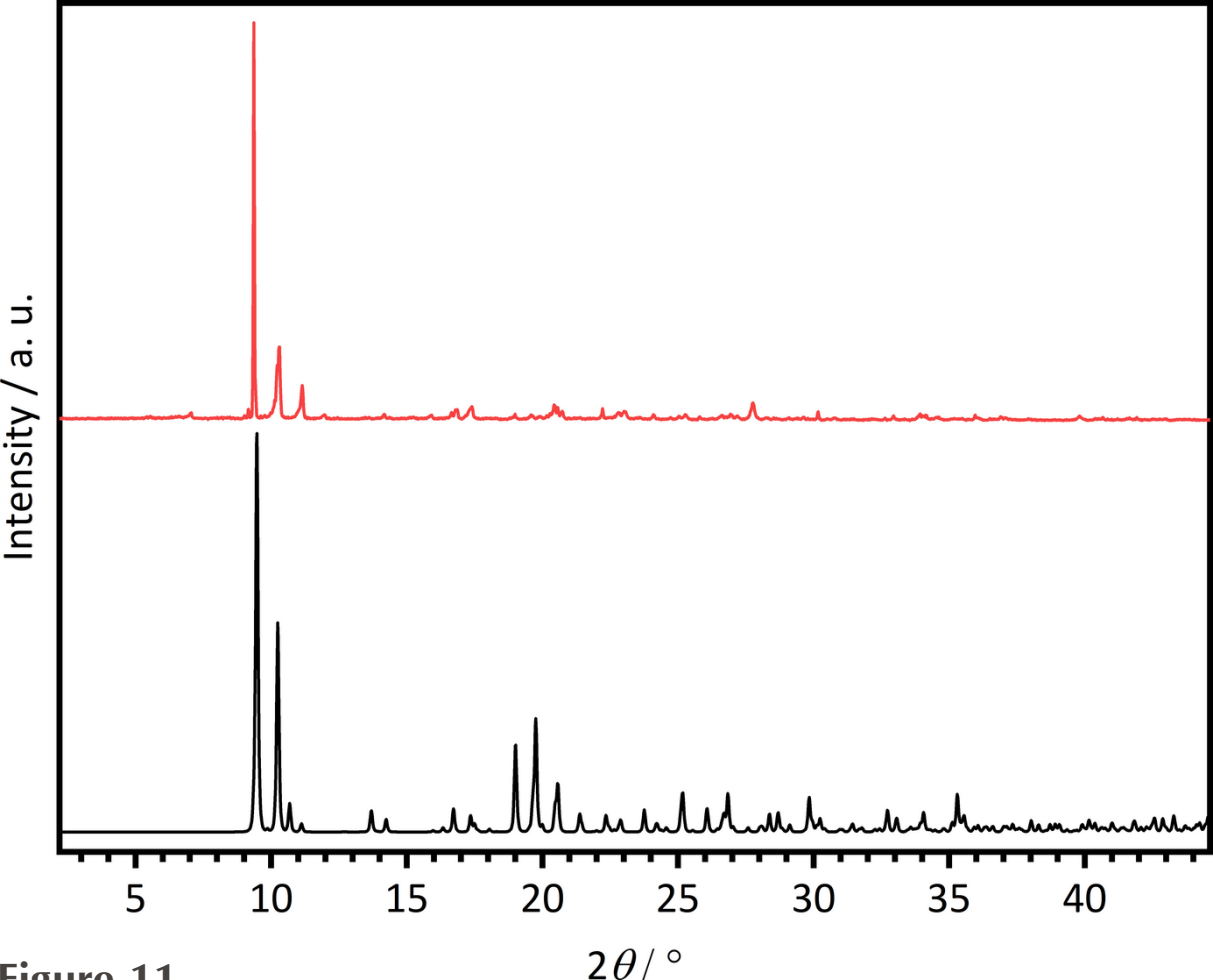
Comparison of the calculated (black) PXRD pattern with the measured one (red) of **1**.

Single crystals of the title compound were obtained by applying the high-throughput method as described in the literature with our custom-made high-throughput setup (Radke *et al.*, 2023 ▸) and were placed in a Memmert UFP400 oven. 36.7 mg (0.21332 mmol) of H_2_TDC (abcr, 97%) were inserted into a 2 mL Teflon vial. 80 µL (0.0640 mmol) of Ce(NO_3_)_3_·6H_2_O [abcr, 99.9% Ce (REO)] in aceto­nitrile (*c* = 0.1333 mol L^−1^), 320 µL of aceto­nitrile and 610 µL of acetic acid were added. The reactor was sealed, placed in an oven and heated to 423 K within 1 h. This temperature was held for 3 h and afterwards the reactor was cooled down to room temperature within 1 h. The reaction mixture was filtered off and washed three times with 3 mL of aceto­nitrile and dried under air. Comparison of the experimental powder pattern of **1** with that calculated from single crystal data reveal that this batch is contaminated with a very small amount of an unknown crystalline phase (Fig. 11 ▸).

**Synthesis of 2**

**Figure 12:**
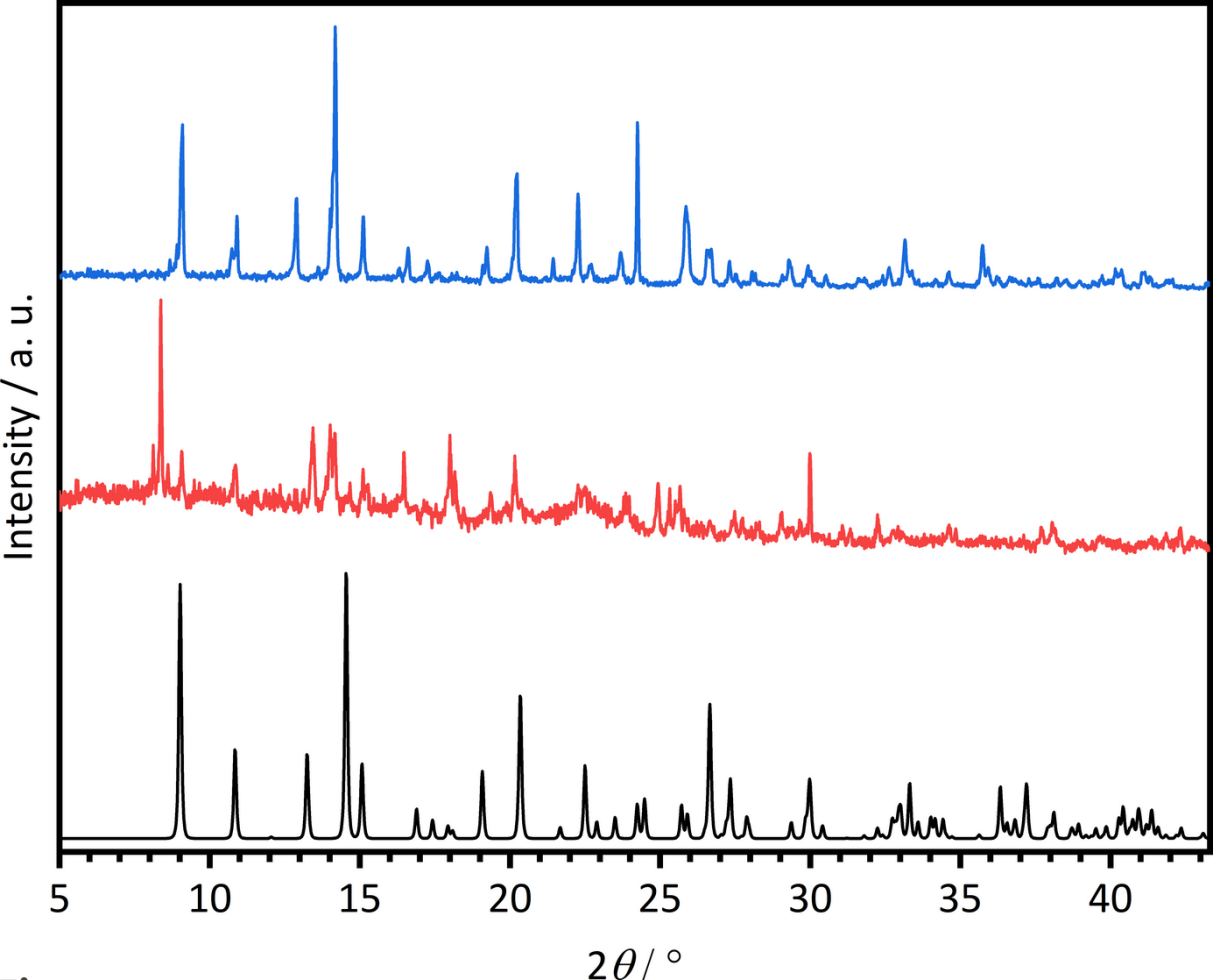
Comparison of the calculated (black) PXRD pattern with the measured ones from sample *A* (red) and sample *B* (blue) of **2**.

Single crystals of the title compound were obtained by applying the high-throughput method mentioned above. Different amounts of H_2_O, EtOH and acetic acid were used to synthesize single crystals suitable for single-crystal X-ray diffraction (sample *A*) and the product containing larger amounts of **2** (sample *B*). In both syntheses, 9.2 mg (0.0533 mmol) of H_2_TDC (abcr, 97%) were inserted into a 2 mL Teflon vial and 400 µL (0.0533 mmol) of Ce(NO_3_)_3_·6H_2_O [abcr, 99.9% Ce (REO)] in H_2_O/EtOH (68:32) (*c* = 0.1333 mol L^−1^) were used. For sample *A*, 131 µL of H_2_O/EtOH (68:32) and 469 µL of acetic acid, and for sample *B*, 470 µL of H_2_O/EtOH (68:32) and 130 µL of acetic acid were added. The reactor was sealed, placed in an oven and heated to 423 K within 24 h. This temperature was held for 192 h and afterwards the reactor was cooled down to room temperature within 48 h. The reaction mixture was filtered off and washed with H_2_O/EtOH (68:32) and dried in air. Compound **2** was not obtained phase pure, as demonstrated by powder X-ray diffraction (Fig. 12 ▸). While sample *A* contained single crystals with large amounts of unidentified byproduct, the majority of sample *B* can be assigned to microcrystalline **2**.

**Experimental details**

The powder X-ray diffraction patterns were collected on a Stoe Stadi P with a MYTHEN2 1K detector and Cu *K*α1 radiation.

## 6. Refinement

Crystal data, data collection and structure refinement details are summarized in Table 5 ▸.

**Refinement of 1**

The C—H H atoms were located in difference maps but were positioned with idealized geometry (methyl H atoms allowed to rotate but not to tip) and were refined isotropically with *U*_iso_(H) = 1.2*U*_eq_(C) (1.5 for methyl H atoms) using a riding model. The O—H H atoms at O24 and O42 were clearly located in difference maps but were finally positioned with idealized geometry, allowed to rotate but not to tip and were refined with *U*_iso_(H) = 1.5*U*_eq_(O) using a riding model. It is noted that some electron density is also found close to O1, which make a hydrogen bond to O42. Because the O⋯O distance between O42 and O1 is relatively short and the C—O distances C41—O42 and C5—O1 are clearly elongated, it cannot be excluded that the H atom is disordered over both sites. Considering these two H atoms, one must assume that compound **1** is a mixed-valance compound, consisting of Ce^III^ and Ce^IV^, but bond-valence calculations using *PLATON* (Spek, 2009 ▸) leads to very similar values for both Ce centers, which deviate only slightly from that expected for Ce^III^. Moreover, because this compound was not obtained as a pure phase, this cannot experimentally proven. Therefore, we assumed that the oxidation state is retained during the synthesis and in this case, one O—H H atom is missing for charge balance. In this context, it is noted that no reasonable electron density was found close to O atoms for carboxyl­ate groups where the C—O distances are slightly different, but for one carboxyl­ate group that shows comparable C—O bond lengths, a reasonable electron density maxima is observed at O33, which would make a hydrogen bond to O22. If an H atom is assigned to this maximum, the corresponding H atom can be refined isotropically, leading to a reasonable temperature factor. This leads to an elongated O—H distance, which indicates that the situation is similar to that between O1 and O42, where the H atom might be disordered. Therefore, the H atom at O33 (H33) was refined with restraints. Finally, it is noted that all carboxyl­ate O atoms except O24 and O42 are coordinated to Ce cations, which might be responsible for the fact that in this case the C—O bond lengths are slightly elongated and not very different. This might also be responsible for the problems in the location of the final H atom and it can also not be excluded that the H atoms are disordered over at least two different sites.

The crystal is twinned by inversion and therefore, a twin-refinement was performed [BASF parameter: 0.491 (4)].

**Refinement of 2**

The C—H H atoms were positioned with idealized geometry (methyl H atoms allowed to rotate but not to tip) and were refined isotropically with *U*_iso_(H) = 1.2*U*_eq_(C) (1.5 for methyl H atoms) using a riding model. The O—H H atoms were located in difference maps and were refined isotropically with *U*_iso_(H) = 1.5*U*_eq_(O) using restraints.

## Supplementary Material

Crystal structure: contains datablock(s) 1, 2. DOI: 10.1107/S2056989025000210/nx2017sup1.cif

Structure factors: contains datablock(s) 1. DOI: 10.1107/S2056989025000210/nx20171sup2.hkl

Structure factors: contains datablock(s) 2. DOI: 10.1107/S2056989025000210/nx20172sup3.hkl

CCDC references: 2415947, 2415946

Additional supporting information:  crystallographic information; 3D view; checkCIF report

## Figures and Tables

**Table 1:** Selected bond lengths (Å) for **1**[Chem scheme1]

Ce1—O1	2.437 (5)	Ce2—O2^iv^	2.481 (5)
Ce1—O4^i^	2.259 (5)	Ce2—O3^ii^	2.527 (5)
Ce1—O11	2.278 (4)	Ce2—O12^iv^	2.518 (4)
Ce1—O13^ii^	2.389 (4)	Ce2—O14^v^	2.538 (4)
Ce1—O21	2.344 (5)	Ce2—O22^vi^	2.502 (5)
Ce1—O32^iii^	2.288 (4)	Ce2—O23	2.555 (4)
Ce1—O34^ii^	2.360 (5)	Ce2—O31	2.342 (5)
Ce1—O41	2.549 (5)	Ce2—O33^v^	2.376 (5)

Symmetry codes: (i) 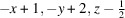; (ii) 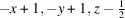; (iii) 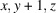; (iv) 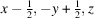; (v) 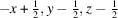; (vi) 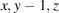.

**Table 2:** Selected bond lengths (Å) for **2**[Chem scheme1]

Ce1—O1	2.422 (3)	Ce1—O5	2.621 (2)
Ce1—O2^i^	2.428 (3)	Ce1—O5^i^	2.466 (2)
Ce1—O3^ii^	2.406 (2)	Ce1—O6	2.551 (3)
Ce1—O4^iii^	2.443 (2)	Ce1—O7	2.503 (3)

Symmetry codes: (i) 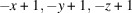; (ii) 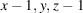; (iii) 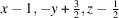.

**Table 3:** Hydrogen-bond geometry (Å, °) for **1**[Chem scheme1]

*D*—H⋯*A*	*D*—H	H⋯*A*	*D*⋯*A*	*D*—H⋯*A*
C12—H12⋯O21	0.95	2.55	3.283 (8)	134
C12—H12⋯O32^iii^	0.95	2.55	3.329 (9)	140
O24—H24⋯O14^v^	0.84	1.77	2.599 (7)	168
O33—H33⋯O22^vii^	0.85 (3)	2.06 (5)	2.871 (7)	159 (11)
C32—H32⋯O12^iv^	0.95	2.55	3.449 (8)	158
O42—H42⋯O1	0.84	1.89	2.671 (8)	155

Symmetry codes: (iii) 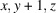; (iv) 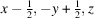; (v) 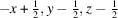; (vii) 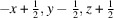.

**Table 4:** Hydrogen-bond geometry (Å, °) for **2**[Chem scheme1]

*D*—H⋯*A*	*D*—H	H⋯*A*	*D*⋯*A*	*D*—H⋯*A*
C8—H8*B*⋯O3^iv^	0.98	2.65	3.497 (5)	145
O7—H7*A*⋯O4^ii^	0.86 (2)	2.02 (2)	2.829 (4)	156 (4)
O7—H7*B*⋯O6^v^	0.85 (2)	1.95 (2)	2.795 (4)	168 (5)

Symmetry codes: (ii) 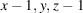; (iv) 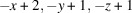; (v) 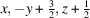.

**Table 5:** Experimental details

	**1**	**2**
Crystal data		
Chemical formula	[Ce_2_(C_6_H_3_O_4_S)_2_(C_6_H_2_OS)_2_(C_2_H_4_O_2_)]	[Ce(C_2_H_3_O_2_)(C_6_H_2_O_4_S)(H_2_O)]
*M*_r_	1022.85	387.32
Crystal system, space group	Orthorhombic, *Pna*2_1_	Monoclinic, *P*2_1_/*c*
Temperature (K)	100	100
*a*, *b*, *c* (Å)	17.91596 (13), 11.08917 (9), 17.25666 (13)	10.0310 (1), 14.6755 (1), 7.6765 (1)
α, β, γ (°)	90, 90, 90	90, 102.354 (1), 90
*V* (Å^3^)	3428.43 (5)	1103.89 (2)
*Z*	4	4
Radiation type	Cu *K*α	Cu *K*α
μ (mm^−1^)	23.23	33.89
Crystal size (mm)	0.08 × 0.08 × 0.03	0.12 × 0.10 × 0.08

Data collection		
Diffractometer	XtaLAB Synergy, Dualflex, HyPix	XtaLAB Synergy, Dualflex, HyPix
Absorption correction	Multi-scan (*CrysAlis PRO*; Rigaku OD, 2023 ▸)	Multi-scan (*CrysAlis PRO*; Rigaku OD, 2023 ▸)
*T*_min_, *T*_max_	0.601, 1.000	0.301, 1.000
No. of measured, independent and observed [*I* > 2σ(*I*)] reflections	29513, 7126, 7101	13515, 2360, 2349
*R*_int_	0.022	0.025
(sin θ/λ)_max_ (Å^−1^)	0.637	0.639

Refinement		
*R*[*F*^2^ > 2σ(*F*^2^)], *wR*(*F*^2^), *S*	0.024, 0.067, 1.05	0.027, 0.077, 1.15
No. of reflections	7126	2360
No. of parameters	458	162
No. of restraints	2	3
H-atom treatment	H atoms treated by a mixture of independent and constrained refinement	H atoms treated by a mixture of independent and constrained refinement
Δρ_max_, Δρ_min_ (e Å^−3^)	1.43, −1.05	1.43, −1.30
Absolute structure	Refined as an inversion twin	–
Absolute structure parameter	0.491 (4)	–

Computer programs: *CrysAlis PRO* (Rigaku OD, 2023 ▸), *SHELXT2014/4* (Sheldrick, 2015*a* ▸), *SHELXL2016/6* (Sheldrick, 2015*b* ▸), *DIAMOND* (Brandenburg & Putz, 1999 ▸), *XP* in *SHELXTL-PC* (Sheldrick, 2008 ▸) and *publCIF* (Westrip, 2010 ▸).

## supplementary crystallographic information

### Poly[(acetic acid)bis(µ-5-carboxythiophene-2-carboxylato)bis(µ-thiophene-2,5-dicarboxylato)dicerium(III)] (1) Crystal data

**Table:**

[Ce_2_(C_6_H_3_O_4_S)_2_(C_6_H_2_OS)_2_(C_2_H_4_O_2_)]	*D*_x_ = 1.982 Mg m^−^^3^
*M**_r_* = 1022.85	Cu *K*α radiation, λ = 1.54184 Å
Orthorhombic, *P**n**a*2_1_	Cell parameters from 25594 reflections
*a* = 17.91596 (13) Å	θ = 2.5–78.6°
*b* = 11.08917 (9) Å	µ = 23.23 mm^−^^1^
*c* = 17.25666 (13) Å	*T* = 100 K
*V* = 3428.43 (5) Å^3^	Block, red
*Z* = 4	0.08 × 0.08 × 0.03 mm
*F*(000) = 1976	

### Poly[(acetic acid)bis(µ-5-carboxythiophene-2-carboxylato)bis(µ-thiophene-2,5-dicarboxylato)dicerium(III)] (1) Data collection

**Table:**

XtaLAB Synergy, Dualflex, HyPix diffractometer	7126 independent reflections
Radiation source: micro-focus sealed X-ray tube, PhotonJet (Cu) X-ray Source	7101 reflections with *I* > 2σ(*I*)
Mirror monochromator	*R*_int_ = 0.022
Detector resolution: 10.0000 pixels mm^-1^	θ_max_ = 79.4°, θ_min_ = 4.7°
ω scans	*h* = −12→20
Absorption correction: multi-scan (CrysAlisPro; Rigaku OD, 2023)	*k* = −13→14
*T*_min_ = 0.601, *T*_max_ = 1.000	*l* = −21→21
29513 measured reflections	

### Poly[(acetic acid)bis(µ-5-carboxythiophene-2-carboxylato)bis(µ-thiophene-2,5-dicarboxylato)dicerium(III)] (1) Refinement

**Table:**

Refinement on *F*^2^	Hydrogen site location: mixed
Least-squares matrix: full	H atoms treated by a mixture of independent and constrained refinement
*R*[*F*^2^ > 2σ(*F*^2^)] = 0.024	*w* = 1/[σ^2^(*F*_o_^2^) + (0.049*P*)^2^ + 4.6448*P*] where *P* = (*F*_o_^2^ + 2*F*_c_^2^)/3
*wR*(*F*^2^) = 0.067	(Δ/σ)_max_ = 0.001
*S* = 1.05	Δρ_max_ = 1.43 e Å^−^^3^
7126 reflections	Δρ_min_ = −1.05 e Å^−^^3^
458 parameters	Absolute structure: Refined as an inversion twin
2 restraints	Absolute structure parameter: 0.491 (4)
Primary atom site location: dual	

### Poly[(acetic acid)bis(µ-5-carboxythiophene-2-carboxylato)bis(µ-thiophene-2,5-dicarboxylato)dicerium(III)] (1) Special details

**Table:**

Geometry. All esds (except the esd in the dihedral angle between two l.s. planes) are estimated using the full covariance matrix. The cell esds are taken into account individually in the estimation of esds in distances, angles and torsion angles; correlations between esds in cell parameters are only used when they are defined by crystal symmetry. An approximate (isotropic) treatment of cell esds is used for estimating esds involving l.s. planes.
Refinement. Refined as a 2-component inversion twin.

### Poly[(acetic acid)bis(µ-5-carboxythiophene-2-carboxylato)bis(µ-thiophene-2,5-dicarboxylato)dicerium(III)] (1) Fractional atomic coordinates and isotropic or equivalent isotropic displacement parameters (Å^2^)

**Table:**

	*x*	*y*	*z*	*U*_iso_*/*U*_eq_	
Ce1	0.46380 (2)	0.80027 (2)	0.28586 (2)	0.01453 (8)	
Ce2	0.18976 (2)	−0.15909 (3)	0.29904 (2)	0.01616 (8)	
O1	0.5601 (3)	0.9287 (4)	0.3419 (3)	0.0244 (9)	
O2	0.6600 (3)	0.8289 (4)	0.3874 (3)	0.0262 (10)	
O3	0.7403 (3)	1.0927 (5)	0.6792 (3)	0.0284 (10)	
O4	0.6311 (3)	1.1855 (5)	0.7001 (3)	0.0324 (11)	
S1	0.68606 (9)	0.97752 (15)	0.52658 (9)	0.0247 (3)	
C1	0.6206 (3)	0.9991 (6)	0.4542 (4)	0.0213 (12)	
C2	0.5756 (4)	1.0967 (6)	0.4703 (4)	0.0273 (14)	
H2	0.537576	1.124358	0.436258	0.033*	
C3	0.5914 (4)	1.1509 (6)	0.5416 (4)	0.0257 (13)	
H3	0.564755	1.218090	0.561729	0.031*	
C4	0.6500 (4)	1.0959 (6)	0.5794 (4)	0.0229 (13)	
C5	0.6152 (4)	0.9125 (6)	0.3899 (4)	0.0231 (12)	
C6	0.6772 (4)	1.1245 (6)	0.6581 (4)	0.0210 (12)	
O11	0.5337 (2)	0.6800 (4)	0.3645 (3)	0.0192 (9)	
O12	0.6233 (2)	0.5628 (4)	0.4117 (3)	0.0242 (9)	
O13	0.5094 (2)	0.3635 (4)	0.7003 (3)	0.0215 (9)	
O14	0.4058 (2)	0.4679 (4)	0.7275 (2)	0.0200 (8)	
S11	0.54976 (8)	0.49323 (13)	0.55758 (8)	0.0202 (3)	
C11	0.5132 (4)	0.5817 (6)	0.4845 (4)	0.0218 (12)	
C12	0.4420 (4)	0.6152 (7)	0.4994 (4)	0.0299 (14)	
H12	0.412376	0.661448	0.464759	0.036*	
C13	0.4165 (4)	0.5740 (7)	0.5722 (4)	0.0301 (15)	
H13	0.368422	0.591469	0.592429	0.036*	
C14	0.4687 (3)	0.5061 (6)	0.6106 (4)	0.0198 (12)	
C15	0.5601 (4)	0.6078 (5)	0.4161 (3)	0.0197 (12)	
C16	0.4612 (3)	0.4414 (6)	0.6848 (4)	0.0172 (12)	
O21	0.3820 (3)	0.6444 (4)	0.3206 (3)	0.0239 (10)	
O22	0.2682 (3)	0.6609 (4)	0.2674 (3)	0.0276 (11)	
O23	0.2292 (3)	0.0610 (4)	0.3147 (3)	0.0299 (11)	
O24	0.1564 (4)	0.1784 (5)	0.2421 (4)	0.0438 (15)	
H24	0.130628	0.115383	0.238411	0.066*	
S21	0.23213 (9)	0.40527 (14)	0.26857 (10)	0.0277 (3)	
C21	0.3077 (4)	0.4717 (6)	0.3126 (4)	0.0276 (14)	
C22	0.3471 (5)	0.3929 (7)	0.3566 (6)	0.044 (2)	
H22	0.390305	0.414617	0.385313	0.053*	
C23	0.3170 (5)	0.2748 (7)	0.3551 (6)	0.044 (2)	
H23	0.337410	0.208507	0.382821	0.053*	
C24	0.2546 (4)	0.2677 (6)	0.3087 (4)	0.0303 (14)	
C25	0.3202 (3)	0.6025 (5)	0.2991 (4)	0.0219 (11)	
C26	0.2124 (4)	0.1592 (5)	0.2890 (5)	0.0287 (14)	
O31	0.2886 (3)	−0.1471 (4)	0.3875 (3)	0.0289 (11)	
O32	0.4117 (3)	−0.1330 (4)	0.3991 (3)	0.0252 (9)	
O33	0.3789 (3)	0.2171 (4)	0.7121 (3)	0.0297 (11)	
H33	0.334 (2)	0.193 (9)	0.717 (7)	0.044*	
O34	0.4761 (3)	0.1068 (5)	0.6798 (3)	0.0279 (10)	
S31	0.41620 (8)	−0.01107 (14)	0.54570 (9)	0.0223 (3)	
C31	0.3376 (3)	−0.0379 (6)	0.4919 (3)	0.0182 (11)	
C32	0.2764 (4)	0.0208 (7)	0.5203 (4)	0.0271 (14)	
H32	0.227951	0.015883	0.498103	0.032*	
C33	0.2940 (4)	0.0900 (6)	0.5868 (4)	0.0265 (14)	
H33A	0.258660	0.137317	0.614346	0.032*	
C34	0.3674 (4)	0.0811 (6)	0.6069 (3)	0.0209 (12)	
C35	0.3462 (4)	−0.1112 (5)	0.4215 (4)	0.0194 (12)	
C36	0.4093 (4)	0.1370 (6)	0.6702 (4)	0.0247 (13)	
O41	0.4239 (3)	1.0183 (5)	0.2643 (3)	0.0365 (12)	
O42	0.5238 (4)	1.1350 (6)	0.2716 (5)	0.0552 (18)	
H42	0.535686	1.084306	0.305602	0.083*	
C41	0.4562 (5)	1.1099 (7)	0.2450 (5)	0.0397 (19)	
C42	0.4262 (6)	1.2017 (9)	0.1916 (7)	0.056 (3)	
H42A	0.460229	1.211191	0.147552	0.084*	
H42B	0.421445	1.278750	0.218924	0.084*	
H42C	0.377096	1.176024	0.172853	0.084*	

### Poly[(acetic acid)bis(µ-5-carboxythiophene-2-carboxylato)bis(µ-thiophene-2,5-dicarboxylato)dicerium(III)] (1) Atomic displacement parameters (Å^2^)

**Table:**

	*U*^11^	*U*^22^	*U*^33^	*U*^12^	*U*^13^	*U*^23^
Ce1	0.02098 (15)	0.01503 (14)	0.00758 (14)	0.00154 (9)	0.00108 (12)	0.00061 (11)
Ce2	0.02058 (15)	0.01835 (14)	0.00956 (14)	−0.00248 (10)	−0.00244 (13)	0.00005 (11)
O1	0.030 (2)	0.025 (2)	0.018 (2)	−0.0008 (18)	−0.0033 (18)	0.0006 (18)
O2	0.032 (3)	0.023 (2)	0.024 (3)	0.0029 (19)	−0.003 (2)	−0.0046 (18)
O3	0.031 (3)	0.033 (2)	0.021 (2)	0.001 (2)	−0.0032 (19)	−0.0059 (19)
O4	0.035 (3)	0.040 (3)	0.022 (3)	0.006 (2)	0.003 (2)	−0.013 (2)
S1	0.0263 (8)	0.0282 (7)	0.0195 (7)	0.0050 (6)	−0.0035 (6)	−0.0081 (6)
C1	0.021 (3)	0.027 (3)	0.015 (3)	−0.002 (2)	0.000 (2)	−0.006 (2)
C2	0.027 (3)	0.030 (3)	0.025 (3)	0.004 (3)	0.001 (3)	−0.003 (3)
C3	0.029 (3)	0.023 (3)	0.025 (3)	0.004 (2)	0.001 (3)	−0.006 (2)
C4	0.025 (3)	0.024 (3)	0.019 (3)	−0.002 (2)	0.003 (2)	−0.005 (2)
C5	0.029 (3)	0.025 (3)	0.015 (3)	−0.007 (3)	0.001 (2)	−0.001 (2)
C6	0.024 (3)	0.021 (3)	0.017 (3)	−0.002 (2)	−0.001 (2)	−0.004 (2)
O11	0.028 (2)	0.0177 (18)	0.012 (2)	0.0019 (16)	0.0016 (17)	0.0038 (17)
O12	0.020 (2)	0.030 (2)	0.022 (2)	0.0043 (18)	0.0020 (16)	0.0052 (18)
O13	0.022 (2)	0.026 (2)	0.016 (2)	0.0053 (18)	0.0021 (17)	0.0087 (17)
O14	0.024 (2)	0.0199 (19)	0.0166 (19)	0.0025 (16)	0.0033 (17)	0.0037 (17)
S11	0.0190 (6)	0.0258 (7)	0.0158 (7)	0.0035 (5)	0.0021 (5)	0.0087 (6)
C11	0.026 (3)	0.023 (3)	0.017 (3)	0.001 (2)	0.006 (2)	0.007 (2)
C12	0.026 (3)	0.039 (4)	0.025 (3)	0.004 (3)	0.003 (3)	0.015 (3)
C13	0.026 (3)	0.042 (4)	0.022 (3)	0.009 (3)	0.006 (3)	0.012 (3)
C14	0.017 (3)	0.027 (3)	0.015 (3)	−0.001 (2)	0.001 (2)	0.010 (2)
C15	0.028 (3)	0.018 (3)	0.014 (3)	−0.004 (2)	0.004 (2)	0.001 (2)
C16	0.017 (3)	0.019 (3)	0.016 (3)	−0.002 (2)	−0.0022 (19)	0.006 (2)
O21	0.026 (2)	0.025 (2)	0.020 (2)	0.0007 (16)	−0.0014 (17)	−0.0063 (17)
O22	0.036 (3)	0.021 (2)	0.025 (3)	−0.0049 (17)	−0.009 (2)	0.0027 (17)
O23	0.034 (2)	0.022 (2)	0.033 (3)	−0.0054 (18)	−0.006 (2)	0.0018 (19)
O24	0.044 (3)	0.022 (2)	0.065 (4)	−0.006 (2)	−0.024 (3)	0.006 (2)
S21	0.0290 (8)	0.0203 (6)	0.0338 (9)	−0.0040 (5)	−0.0056 (6)	0.0016 (6)
C21	0.027 (3)	0.022 (3)	0.033 (4)	0.001 (2)	−0.002 (2)	−0.001 (3)
C22	0.044 (4)	0.024 (3)	0.064 (6)	−0.007 (3)	−0.022 (4)	0.003 (3)
C23	0.049 (5)	0.023 (3)	0.060 (6)	−0.005 (3)	−0.017 (4)	0.005 (4)
C24	0.033 (3)	0.026 (3)	0.032 (4)	−0.003 (3)	−0.002 (3)	−0.001 (3)
C25	0.025 (3)	0.021 (3)	0.020 (3)	−0.002 (2)	0.000 (2)	−0.003 (3)
C26	0.030 (3)	0.025 (3)	0.032 (4)	−0.003 (2)	0.000 (3)	0.000 (3)
O31	0.028 (2)	0.032 (3)	0.027 (3)	0.0014 (19)	−0.011 (2)	−0.013 (2)
O32	0.028 (2)	0.029 (2)	0.018 (2)	−0.0017 (19)	0.0069 (18)	−0.0082 (19)
O33	0.042 (3)	0.027 (2)	0.020 (2)	−0.008 (2)	0.012 (2)	−0.0117 (19)
O34	0.027 (2)	0.036 (3)	0.021 (2)	−0.005 (2)	−0.0036 (18)	−0.007 (2)
S31	0.0204 (7)	0.0285 (7)	0.0178 (7)	0.0003 (5)	−0.0019 (5)	−0.0110 (6)
C31	0.020 (3)	0.023 (3)	0.012 (3)	−0.001 (2)	0.000 (2)	−0.005 (2)
C32	0.019 (3)	0.041 (4)	0.021 (3)	0.001 (3)	−0.004 (2)	−0.013 (3)
C33	0.029 (3)	0.027 (3)	0.023 (3)	0.004 (3)	0.005 (3)	−0.014 (3)
C34	0.026 (3)	0.024 (3)	0.013 (3)	0.000 (2)	0.000 (2)	−0.009 (2)
C35	0.024 (3)	0.019 (3)	0.016 (3)	0.001 (2)	−0.005 (2)	−0.001 (2)
C36	0.033 (4)	0.025 (3)	0.015 (3)	−0.003 (3)	0.002 (3)	−0.008 (2)
O41	0.035 (3)	0.033 (3)	0.042 (3)	0.005 (2)	−0.001 (2)	−0.001 (2)
O42	0.061 (4)	0.038 (3)	0.067 (5)	−0.013 (3)	−0.015 (3)	0.012 (3)
C41	0.050 (5)	0.030 (4)	0.039 (5)	0.000 (3)	−0.009 (3)	−0.001 (3)
C42	0.069 (7)	0.040 (5)	0.060 (7)	0.006 (4)	−0.001 (5)	0.015 (4)

### Poly[(acetic acid)bis(µ-5-carboxythiophene-2-carboxylato)bis(µ-thiophene-2,5-dicarboxylato)dicerium(III)] (1) Geometric parameters (Å, º)

**Table:**

Ce1—O1	2.437 (5)	C13—H13	0.9500
Ce1—O4^i^	2.259 (5)	C13—C14	1.371 (9)
Ce1—O11	2.278 (4)	C14—C16	1.474 (8)
Ce1—O13^ii^	2.389 (4)	O21—C25	1.257 (7)
Ce1—O21	2.344 (5)	O22—C25	1.259 (8)
Ce1—O32^iii^	2.288 (4)	O23—C26	1.214 (8)
Ce1—O34^ii^	2.360 (5)	O24—H24	0.8400
Ce1—O41	2.549 (5)	O24—C26	1.306 (9)
Ce2—O2^iv^	2.481 (5)	S21—C21	1.718 (7)
Ce2—O3^ii^	2.527 (5)	S21—C24	1.723 (7)
Ce2—O12^iv^	2.518 (4)	C21—C22	1.356 (11)
Ce2—O14^v^	2.538 (4)	C21—C25	1.486 (8)
Ce2—O22^vi^	2.502 (5)	C22—H22	0.9500
Ce2—O23	2.555 (4)	C22—C23	1.416 (10)
Ce2—O31	2.342 (5)	C23—H23	0.9500
Ce2—O33^v^	2.376 (5)	C23—C24	1.378 (11)
O1—C5	1.301 (8)	C24—C26	1.462 (9)
O2—C5	1.226 (8)	O31—C35	1.252 (8)
O3—C6	1.240 (8)	O32—C35	1.260 (8)
O4—C6	1.290 (8)	O33—H33	0.85 (3)
S1—C1	1.730 (6)	O33—C36	1.268 (8)
S1—C4	1.724 (6)	O34—C36	1.255 (9)
C1—C2	1.377 (9)	S31—C31	1.713 (6)
C1—C5	1.470 (8)	S31—C34	1.710 (6)
C2—H2	0.9500	C31—C32	1.366 (9)
C2—C3	1.400 (9)	C31—C35	1.469 (8)
C3—H3	0.9500	C32—H32	0.9500
C3—C4	1.378 (10)	C32—C33	1.417 (9)
C4—C6	1.478 (9)	C33—H33A	0.9500
O11—C15	1.287 (8)	C33—C34	1.363 (10)
O12—C15	1.239 (8)	C34—C36	1.463 (8)
O13—C16	1.249 (7)	O41—C41	1.215 (10)
O14—C16	1.270 (7)	O42—H42	0.8400
S11—C11	1.727 (6)	O42—C41	1.324 (10)
S11—C14	1.722 (6)	C41—C42	1.475 (12)
C11—C12	1.354 (10)	C42—H42A	0.9800
C11—C15	1.478 (8)	C42—H42B	0.9800
C12—H12	0.9500	C42—H42C	0.9800
C12—C13	1.412 (9)		
			
O1—Ce1—O41	72.69 (16)	C16—O13—Ce1^viii^	142.8 (4)
O4^i^—Ce1—O1	138.75 (17)	C16—O14—Ce2^x^	132.9 (4)
O4^i^—Ce1—O11	147.65 (18)	C14—S11—C11	91.2 (3)
O4^i^—Ce1—O13^ii^	78.45 (19)	C12—C11—S11	112.0 (5)
O4^i^—Ce1—O21	75.46 (18)	C12—C11—C15	129.4 (6)
O4^i^—Ce1—O32^iii^	103.31 (19)	C15—C11—S11	118.6 (5)
O4^i^—Ce1—O34^ii^	78.76 (18)	C11—C12—H12	123.7
O4^i^—Ce1—O41	68.09 (19)	C11—C12—C13	112.6 (6)
O11—Ce1—O1	73.52 (16)	C13—C12—H12	123.7
O11—Ce1—O13^ii^	79.19 (16)	C12—C13—H13	123.6
O11—Ce1—O21	76.09 (16)	C14—C13—C12	112.8 (6)
O11—Ce1—O32^iii^	84.53 (17)	C14—C13—H13	123.6
O11—Ce1—O34^ii^	117.81 (16)	C13—C14—S11	111.3 (5)
O11—Ce1—O41	142.83 (17)	C13—C14—C16	128.7 (5)
O13^ii^—Ce1—O1	123.16 (16)	C16—C14—S11	119.9 (4)
O13^ii^—Ce1—O41	133.41 (17)	O11—C15—C11	117.7 (6)
O21—Ce1—O1	140.50 (16)	O12—C15—O11	123.0 (6)
O21—Ce1—O13^ii^	73.96 (16)	O12—C15—C11	119.3 (5)
O21—Ce1—O34^ii^	143.69 (17)	O13—C16—O14	125.1 (6)
O21—Ce1—O41	124.15 (16)	O13—C16—C14	117.4 (5)
O32^iii^—Ce1—O1	76.14 (16)	O14—C16—C14	117.5 (5)
O32^iii^—Ce1—O13^ii^	148.81 (17)	C25—O21—Ce1	138.4 (4)
O32^iii^—Ce1—O21	76.40 (16)	C25—O22—Ce2^iii^	136.9 (4)
O32^iii^—Ce1—O34^ii^	134.98 (18)	C26—O23—Ce2	138.5 (5)
O32^iii^—Ce1—O41	72.77 (18)	C26—O24—H24	109.5
O34^ii^—Ce1—O1	74.31 (16)	C21—S21—C24	91.0 (3)
O34^ii^—Ce1—O13^ii^	76.18 (17)	C22—C21—S21	112.4 (5)
O34^ii^—Ce1—O41	66.45 (18)	C22—C21—C25	129.7 (7)
O2^iv^—Ce2—O3^ii^	146.16 (16)	C25—C21—S21	117.9 (5)
O2^iv^—Ce2—O12^iv^	75.25 (16)	C21—C22—H22	123.6
O2^iv^—Ce2—O14^v^	125.11 (15)	C21—C22—C23	112.8 (7)
O2^iv^—Ce2—O22^vi^	69.48 (17)	C23—C22—H22	123.6
O2^iv^—Ce2—O23	136.00 (16)	C22—C23—H23	124.1
O3^ii^—Ce2—O14^v^	76.98 (15)	C24—C23—C22	111.8 (7)
O3^ii^—Ce2—O23	70.81 (17)	C24—C23—H23	124.1
O12^iv^—Ce2—O3^ii^	137.97 (16)	C23—C24—S21	111.9 (5)
O12^iv^—Ce2—O14^v^	79.70 (15)	C23—C24—C26	127.0 (7)
O12^iv^—Ce2—O23	69.15 (16)	C26—C24—S21	120.9 (5)
O14^v^—Ce2—O23	73.03 (14)	O21—C25—O22	126.2 (5)
O22^vi^—Ce2—O3^ii^	77.01 (16)	O21—C25—C21	116.5 (6)
O22^vi^—Ce2—O12^iv^	140.61 (16)	O22—C25—C21	117.3 (5)
O22^vi^—Ce2—O14^v^	135.69 (15)	O23—C26—O24	124.3 (6)
O22^vi^—Ce2—O23	129.04 (15)	O23—C26—C24	121.6 (7)
O31—Ce2—O2^iv^	78.76 (18)	O24—C26—C24	114.0 (6)
O31—Ce2—O3^ii^	98.17 (17)	C35—O31—Ce2	161.2 (5)
O31—Ce2—O12^iv^	80.23 (17)	C35—O32—Ce1^vi^	134.8 (4)
O31—Ce2—O14^v^	142.75 (15)	Ce2^x^—O33—H33	68 (7)
O31—Ce2—O22^vi^	76.28 (16)	C36—O33—Ce2^x^	170.6 (5)
O31—Ce2—O23	70.59 (16)	C36—O33—H33	104 (7)
O31—Ce2—O33^v^	146.68 (17)	C36—O34—Ce1^viii^	114.9 (4)
O33^v^—Ce2—O2^iv^	80.68 (18)	C34—S31—C31	91.0 (3)
O33^v^—Ce2—O3^ii^	84.75 (18)	C32—C31—S31	112.5 (5)
O33^v^—Ce2—O12^iv^	119.17 (16)	C32—C31—C35	130.1 (6)
O33^v^—Ce2—O14^v^	70.38 (15)	C35—C31—S31	117.2 (5)
O33^v^—Ce2—O22^vi^	72.06 (16)	C31—C32—H32	124.1
O33^v^—Ce2—O23	139.62 (16)	C31—C32—C33	111.7 (6)
C5—O1—Ce1	135.2 (4)	C33—C32—H32	124.1
C5—O2—Ce2^vii^	137.4 (5)	C32—C33—H33A	123.8
C6—O3—Ce2^viii^	127.5 (4)	C34—C33—C32	112.5 (6)
C6—O4—Ce1^ix^	152.4 (5)	C34—C33—H33A	123.8
C4—S1—C1	91.3 (3)	C33—C34—S31	112.3 (5)
C2—C1—S1	111.1 (5)	C33—C34—C36	130.8 (6)
C2—C1—C5	128.8 (6)	C36—C34—S31	116.9 (5)
C5—C1—S1	119.9 (5)	O31—C35—O32	124.2 (6)
C1—C2—H2	123.3	O31—C35—C31	118.5 (6)
C1—C2—C3	113.4 (6)	O32—C35—C31	117.3 (6)
C3—C2—H2	123.3	O33—C36—C34	120.2 (6)
C2—C3—H3	123.8	O34—C36—O33	121.4 (6)
C4—C3—C2	112.3 (6)	O34—C36—C34	118.3 (6)
C4—C3—H3	123.8	C41—O41—Ce1	134.3 (5)
C3—C4—S1	111.9 (5)	C41—O42—H42	109.5
C3—C4—C6	126.2 (6)	O41—C41—O42	121.1 (8)
C6—C4—S1	121.7 (5)	O41—C41—C42	125.0 (8)
O1—C5—C1	116.1 (6)	O42—C41—C42	113.8 (8)
O2—C5—O1	125.3 (6)	C41—C42—H42A	109.5
O2—C5—C1	118.5 (6)	C41—C42—H42B	109.5
O3—C6—O4	124.7 (6)	C41—C42—H42C	109.5
O3—C6—C4	120.6 (6)	H42A—C42—H42B	109.5
O4—C6—C4	114.7 (6)	H42A—C42—H42C	109.5
C15—O11—Ce1	168.0 (4)	H42B—C42—H42C	109.5
C15—O12—Ce2^vii^	107.9 (4)		

Symmetry codes: (i) −*x*+1, −*y*+2, *z*−1/2; (ii) −*x*+1, −*y*+1, *z*−1/2; (iii) *x*, *y*+1, *z*; (iv) *x*−1/2, −*y*+1/2, *z*; (v) −*x*+1/2, *y*−1/2, *z*−1/2; (vi) *x*, *y*−1, *z*; (vii) *x*+1/2, −*y*+1/2, *z*; (viii) −*x*+1, −*y*+1, *z*+1/2; (ix) −*x*+1, −*y*+2, *z*+1/2; (x) −*x*+1/2, *y*+1/2, *z*+1/2.

### Poly[(acetic acid)bis(µ-5-carboxythiophene-2-carboxylato)bis(µ-thiophene-2,5-dicarboxylato)dicerium(III)] (1) Hydrogen-bond geometry (Å, º)

**Table:**

*D*—H···*A*	*D*—H	H···*A*	*D*···*A*	*D*—H···*A*
C12—H12···O21	0.95	2.55	3.283 (8)	134
C12—H12···O32^iii^	0.95	2.55	3.329 (9)	140
O24—H24···O14^v^	0.84	1.77	2.599 (7)	168
O33—H33···O22^xi^	0.85 (3)	2.06 (5)	2.871 (7)	159 (11)
C32—H32···O12^iv^	0.95	2.55	3.449 (8)	158
O42—H42···O1	0.84	1.89	2.671 (8)	155

Symmetry codes: (iii) *x*, *y*+1, *z*; (iv) *x*−1/2, −*y*+1/2, *z*; (v) −*x*+1/2, *y*−1/2, *z*−1/2; (xi) −*x*+1/2, *y*−1/2, *z*+1/2.

### Poly[(µ-acetato)aqua(µ4-thiophene-2,5-dicarboxylato)cerium(III)] (2) Crystal data

**Table:**

[Ce(C_2_H_3_O_2_)(C_6_H_2_O_4_S)(H_2_O)]	*F*(000) = 740
*M**_r_* = 387.32	*D*_x_ = 2.330 Mg m^−^^3^
Monoclinic, *P*2_1_/*c*	Cu *K*α radiation, λ = 1.54184 Å
*a* = 10.0310 (1) Å	Cell parameters from 10498 reflections
*b* = 14.6755 (1) Å	θ = 3.1–79.9°
*c* = 7.6765 (1) Å	µ = 33.89 mm^−^^1^
β = 102.354 (1)°	*T* = 100 K
*V* = 1103.89 (2) Å^3^	Block, colorless
*Z* = 4	0.12 × 0.10 × 0.08 mm

### Poly[(µ-acetato)aqua(µ4-thiophene-2,5-dicarboxylato)cerium(III)] (2) Data collection

**Table:**

XtaLAB Synergy, Dualflex, HyPix diffractometer	2360 independent reflections
Radiation source: micro-focus sealed X-ray tube, PhotonJet (Cu) X-ray Source	2349 reflections with *I* > 2σ(*I*)
Mirror monochromator	*R*_int_ = 0.025
Detector resolution: 10.0000 pixels mm^-1^	θ_max_ = 80.3°, θ_min_ = 4.5°
ω scans	*h* = −12→10
Absorption correction: multi-scan (CrysAlisPro; Rigaku OD, 2023)	*k* = −18→18
*T*_min_ = 0.301, *T*_max_ = 1.000	*l* = −9→9
13515 measured reflections	

### Poly[(µ-acetato)aqua(µ4-thiophene-2,5-dicarboxylato)cerium(III)] (2) Refinement

**Table:**

Refinement on *F*^2^	Hydrogen site location: mixed
Least-squares matrix: full	H atoms treated by a mixture of independent and constrained refinement
*R*[*F*^2^ > 2σ(*F*^2^)] = 0.027	*w* = 1/[σ^2^(*F*_o_^2^) + (0.0508*P*)^2^ + 2.3818*P*] where *P* = (*F*_o_^2^ + 2*F*_c_^2^)/3
*wR*(*F*^2^) = 0.077	(Δ/σ)_max_ = 0.002
*S* = 1.15	Δρ_max_ = 1.43 e Å^−^^3^
2360 reflections	Δρ_min_ = −1.30 e Å^−^^3^
162 parameters	Extinction correction: *SHELXL2016/6* (Sheldrick, 2015b), Fc^*^=kFc[1+0.001xFc^2^λ^3^/sin(2θ)]^-1/4^
3 restraints	Extinction coefficient: 0.00097 (10)
Primary atom site location: dual	

### Poly[(µ-acetato)aqua(µ4-thiophene-2,5-dicarboxylato)cerium(III)] (2) Special details

**Table:**

Geometry. All esds (except the esd in the dihedral angle between two l.s. planes) are estimated using the full covariance matrix. The cell esds are taken into account individually in the estimation of esds in distances, angles and torsion angles; correlations between esds in cell parameters are only used when they are defined by crystal symmetry. An approximate (isotropic) treatment of cell esds is used for estimating esds involving l.s. planes.

### Poly[(µ-acetato)aqua(µ4-thiophene-2,5-dicarboxylato)cerium(III)] (2) Fractional atomic coordinates and isotropic or equivalent isotropic displacement parameters (Å^2^)

**Table:**

	*x*	*y*	*z*	*U*_iso_*/*U*_eq_	
Ce1	0.39777 (2)	0.61197 (2)	0.39322 (2)	0.00537 (11)	
S1	0.97823 (9)	0.62456 (6)	0.89764 (12)	0.00924 (18)	
C1	0.8335 (3)	0.6693 (2)	0.7640 (5)	0.0091 (7)	
C2	0.8385 (4)	0.7628 (3)	0.7551 (5)	0.0120 (7)	
H2	0.766359	0.799189	0.690202	0.014*	
C3	0.9626 (4)	0.7987 (2)	0.8530 (5)	0.0123 (7)	
H3	0.984488	0.861789	0.859212	0.015*	
C4	1.0481 (3)	0.7316 (2)	0.9386 (5)	0.0081 (6)	
C5	0.7227 (4)	0.6083 (2)	0.6703 (5)	0.0102 (7)	
O1	0.6225 (3)	0.64614 (19)	0.5702 (4)	0.0139 (5)	
O2	0.7387 (3)	0.52357 (18)	0.6960 (4)	0.0140 (5)	
C6	1.1848 (3)	0.7417 (2)	1.0561 (4)	0.0068 (6)	
O3	1.2357 (2)	0.67297 (17)	1.1434 (3)	0.0102 (5)	
O4	1.2436 (2)	0.81927 (17)	1.0629 (3)	0.0095 (5)	
O5	0.5659 (3)	0.48401 (17)	0.3371 (3)	0.0110 (5)	
O6	0.5347 (3)	0.59880 (18)	0.1501 (4)	0.0122 (5)	
C7	0.6028 (3)	0.5302 (2)	0.2133 (5)	0.0077 (6)	
C8	0.7282 (4)	0.5017 (3)	0.1529 (5)	0.0136 (7)	
H8A	0.743427	0.542620	0.058242	0.016*	
H8B	0.716894	0.439144	0.107223	0.016*	
H8C	0.806844	0.504409	0.253655	0.016*	
O7	0.4453 (3)	0.77851 (18)	0.3711 (3)	0.0121 (5)	
H7A	0.387 (4)	0.807 (3)	0.291 (5)	0.018*	
H7B	0.484 (5)	0.816 (3)	0.452 (5)	0.018*	

### Poly[(µ-acetato)aqua(µ4-thiophene-2,5-dicarboxylato)cerium(III)] (2) Atomic displacement parameters (Å^2^)

**Table:**

	*U*^11^	*U*^22^	*U*^33^	*U*^12^	*U*^13^	*U*^23^
Ce1	0.00288 (14)	0.00494 (15)	0.00770 (14)	0.00049 (5)	−0.00018 (9)	0.00042 (5)
S1	0.0047 (4)	0.0073 (4)	0.0142 (4)	−0.0007 (3)	−0.0013 (3)	0.0008 (3)
C1	0.0040 (15)	0.0136 (17)	0.0087 (15)	−0.0003 (12)	−0.0006 (12)	−0.0001 (12)
C2	0.0061 (16)	0.0130 (18)	0.0149 (18)	0.0005 (12)	−0.0021 (13)	0.0017 (13)
C3	0.0088 (15)	0.0073 (16)	0.0182 (17)	0.0007 (12)	−0.0025 (13)	0.0023 (13)
C4	0.0050 (15)	0.0097 (16)	0.0086 (15)	−0.0022 (12)	−0.0008 (12)	0.0006 (12)
C5	0.0076 (17)	0.0100 (18)	0.0129 (18)	−0.0026 (11)	0.0019 (14)	−0.0007 (12)
O1	0.0087 (11)	0.0122 (13)	0.0177 (13)	−0.0028 (10)	−0.0041 (10)	0.0027 (11)
O2	0.0095 (12)	0.0116 (13)	0.0173 (13)	−0.0013 (9)	−0.0051 (10)	0.0021 (10)
C6	0.0038 (15)	0.0112 (16)	0.0052 (15)	−0.0005 (12)	0.0006 (12)	−0.0011 (12)
O3	0.0071 (11)	0.0121 (12)	0.0091 (11)	0.0001 (9)	−0.0030 (9)	0.0018 (9)
O4	0.0071 (11)	0.0082 (12)	0.0122 (11)	−0.0025 (9)	0.0000 (9)	−0.0007 (9)
O5	0.0106 (12)	0.0126 (12)	0.0107 (11)	0.0020 (9)	0.0039 (9)	0.0030 (9)
O6	0.0123 (13)	0.0101 (11)	0.0148 (13)	0.0026 (10)	0.0046 (10)	0.0033 (10)
C7	0.0074 (15)	0.0065 (15)	0.0086 (14)	−0.0010 (12)	0.0006 (12)	0.0023 (12)
C8	0.0126 (17)	0.0115 (17)	0.0195 (17)	−0.0005 (13)	0.0093 (14)	−0.0016 (14)
O7	0.0131 (13)	0.0106 (12)	0.0102 (12)	−0.0024 (10)	−0.0030 (10)	−0.0006 (9)

### Poly[(µ-acetato)aqua(µ4-thiophene-2,5-dicarboxylato)cerium(III)] (2) Geometric parameters (Å, º)

**Table:**

Ce1—Ce1^i^	4.0320 (3)	C3—H3	0.9500
Ce1—O1	2.422 (3)	C3—C4	1.377 (5)
Ce1—O2^i^	2.428 (3)	C4—C6	1.479 (4)
Ce1—O3^ii^	2.406 (2)	C5—O1	1.257 (5)
Ce1—O4^iii^	2.443 (2)	C5—O2	1.263 (4)
Ce1—O5	2.621 (2)	C6—O3	1.258 (4)
Ce1—O5^i^	2.466 (2)	C6—O4	1.277 (4)
Ce1—O6	2.551 (3)	O5—C7	1.285 (4)
Ce1—C7	2.965 (4)	O6—C7	1.254 (4)
Ce1—O7	2.503 (3)	C7—C8	1.491 (5)
S1—C1	1.719 (3)	C8—H8A	0.9800
S1—C4	1.722 (4)	C8—H8B	0.9800
C1—C2	1.375 (5)	C8—H8C	0.9800
C1—C5	1.488 (5)	O7—H7A	0.861 (19)
C2—H2	0.9500	O7—H7B	0.854 (19)
C2—C3	1.411 (5)		
			
O1—Ce1—Ce1^i^	67.00 (6)	O7—Ce1—C7	101.68 (9)
O1—Ce1—O2^i^	136.75 (9)	C1—S1—C4	91.09 (17)
O1—Ce1—O4^iii^	103.58 (9)	C2—C1—S1	112.1 (3)
O1—Ce1—O5	71.71 (9)	C2—C1—C5	127.4 (3)
O1—Ce1—O5^i^	72.22 (9)	C5—C1—S1	120.4 (3)
O1—Ce1—O6	81.02 (10)	C1—C2—H2	123.7
O1—Ce1—C7	71.95 (10)	C1—C2—C3	112.5 (3)
O1—Ce1—O7	70.61 (9)	C3—C2—H2	123.7
O2^i^—Ce1—Ce1^i^	69.76 (6)	C2—C3—H3	124.0
O2^i^—Ce1—O4^iii^	96.07 (9)	C4—C3—C2	112.0 (3)
O2^i^—Ce1—O5^i^	75.06 (9)	C4—C3—H3	124.0
O2^i^—Ce1—O5	73.16 (8)	C3—C4—S1	112.2 (3)
O2^i^—Ce1—O6	95.20 (9)	C3—C4—C6	128.4 (3)
O2^i^—Ce1—C7	86.69 (9)	C6—C4—S1	119.4 (3)
O2^i^—Ce1—O7	152.22 (8)	O1—C5—C1	116.5 (3)
O3^ii^—Ce1—Ce1^i^	145.65 (6)	O1—C5—O2	126.1 (3)
O3^ii^—Ce1—O1	139.39 (9)	O2—C5—C1	117.4 (3)
O3^ii^—Ce1—O2^i^	80.56 (8)	C5—O1—Ce1	141.1 (2)
O3^ii^—Ce1—O4^iii^	82.77 (8)	C5—O2—Ce1^i^	135.8 (2)
O3^ii^—Ce1—O5^i^	146.62 (8)	O3—C6—C4	117.5 (3)
O3^ii^—Ce1—O5	118.98 (8)	O3—C6—O4	124.2 (3)
O3^ii^—Ce1—O6	79.80 (9)	O4—C6—C4	118.3 (3)
O3^ii^—Ce1—C7	101.36 (9)	C6—O3—Ce1^iv^	148.4 (2)
O3^ii^—Ce1—O7	71.86 (8)	C6—O4—Ce1^v^	133.3 (2)
O4^iii^—Ce1—Ce1^i^	116.42 (6)	Ce1^i^—O5—Ce1	104.82 (9)
O4^iii^—Ce1—O5	152.47 (8)	C7—O5—Ce1^i^	155.3 (2)
O4^iii^—Ce1—O5^i^	77.58 (8)	C7—O5—Ce1	92.3 (2)
O4^iii^—Ce1—O6	157.36 (8)	C7—O6—Ce1	96.4 (2)
O4^iii^—Ce1—C7	175.38 (9)	O5—C7—Ce1	62.02 (18)
O4^iii^—Ce1—O7	77.54 (9)	O5—C7—C8	118.5 (3)
O5^i^—Ce1—Ce1^i^	38.93 (6)	O6—C7—Ce1	58.74 (19)
O5—Ce1—Ce1^i^	36.25 (5)	O6—C7—O5	119.4 (3)
O5^i^—Ce1—O5	75.18 (9)	O6—C7—C8	122.1 (3)
O5^i^—Ce1—O6	124.42 (8)	C8—C7—Ce1	166.9 (2)
O5—Ce1—C7	25.65 (9)	C7—C8—H8A	109.5
O5^i^—Ce1—C7	99.64 (9)	C7—C8—H8B	109.5
O5^i^—Ce1—O7	128.22 (8)	C7—C8—H8C	109.5
O6—Ce1—Ce1^i^	85.93 (6)	H8A—C8—H8B	109.5
O6—Ce1—O5	50.13 (8)	H8A—C8—H8C	109.5
O6—Ce1—C7	24.85 (9)	H8B—C8—H8C	109.5
C7—Ce1—Ce1^i^	61.08 (6)	Ce1—O7—H7A	114 (3)
O7—Ce1—Ce1^i^	137.38 (6)	Ce1—O7—H7B	129 (3)
O7—Ce1—O5	123.36 (8)	H7A—O7—H7B	111 (4)
O7—Ce1—O6	83.35 (9)		

Symmetry codes: (i) −*x*+1, −*y*+1, −*z*+1; (ii) *x*−1, *y*, *z*−1; (iii) *x*−1, −*y*+3/2, *z*−1/2; (iv) *x*+1, *y*, *z*+1; (v) *x*+1, −*y*+3/2, *z*+1/2.

### Poly[(µ-acetato)aqua(µ4-thiophene-2,5-dicarboxylato)cerium(III)] (2) Hydrogen-bond geometry (Å, º)

**Table:**

*D*—H···*A*	*D*—H	H···*A*	*D*···*A*	*D*—H···*A*
C8—H8*B*···O3^vi^	0.98	2.65	3.497 (5)	145
O7—H7*A*···O4^ii^	0.86 (2)	2.02 (2)	2.829 (4)	156 (4)
O7—H7*B*···O6^vii^	0.85 (2)	1.95 (2)	2.795 (4)	168 (5)

Symmetry codes: (ii) *x*−1, *y*, *z*−1; (vi) −*x*+2, −*y*+1, −*z*+1; (vii) *x*, −*y*+3/2, *z*+1/2.
